# Longitudinal Monitoring of Mono- and Coinfections Involving Primary Porcine Reproductive Viruses (PCV2, PPV1, and PRRSV) as Well as Emerging Viruses (PCV3, PCV4, and nPPVs) in Primiparous and Multiparous Sows and Their Litters

**DOI:** 10.3390/pathogens14060573

**Published:** 2025-06-07

**Authors:** Diana S. Vargas-Bermudez, Gina Polo, Jose Dario Mogollon, Jairo Jaime

**Affiliations:** 1 Universidad Nacional de Colombia, Sede Bogotá, Facultad de Medicina Veterinaria y de Zootecnia, Departamento de Salud Animal, Centro de Investigación en Infectología e Inmunología Veterinaria–CI3V, Cra. 30 # 45-03, Bogotá 111321, Colombia; dsvargasb@unal.edu.co (D.S.V.-B.); josedmogollon@yahoo.es (J.D.M.); 2 Pontificia Universidad Javeriana, Instituto de Salud Pública, Cra. 7 # 40-62, Bogotá 110231, Colombia; pologp@javeriana.edu.co

**Keywords:** porcine reproductive failure (PRF), SMEDI, PCV2, PCV3, PPV1, PRRSV, nPPVs, coinfections

## Abstract

Porcine reproductive failure (PRF) has multiple etiological origins, primarily involving the viruses PCV2, PPV1, and PRRSV. Some emerging viruses, such as PCV3, PCV4, and novel parvoviruses (nPPVs), have also been suggested as contributors. In this study, we longitudinally evaluated 40 healthy sows (20 gilts and 20 multiparous sows) over three phases: pregnancy (PP), farrowing (FP), and their litters during lactation (LP). We detected viruses through PCR and serology in mono- and coinfections. The results showed that primary viruses were present during all three phases, with PCV2 being the most frequently detected. PCV3 positivity was highest at the time of insemination, and PPV1 was found in all. Additionally, PPV1-positive fetuses and pre-suckling piglets were identified, indicating vertical transmission. PRRSV was also present in an unstable herd, with the PRRSV2 lineage A detected and evidence of vertical transmission. The majority of coinfections were either dual or triple. The most common coinfections in the PP and LP were PCV2/PPV1 and PCV2/PCV3, while in the FF, PCV2/PPV1 and PCV2/PRRSV predominated. Notably, coinfection PCV2/PPV1 impacted the replication of PCV2. In contrast, the likelihood of detecting PRRSV decreased in fetuses coinfected with PRRSV and either PCV2, PCV3, or PPV1. The detected viruses exhibited low viral loads, indicating subclinical infections. Therefore, we propose recognizing a subclinical presentation of PRF and establishing criteria to differentiate between this and symptomatic reproductive disease.

## 1. Introduction

Porcine reproductive failure (PRF) is a complex syndrome that causes significant economic losses to the swine industry worldwide [1,2]. The main clinical signs of PRF in both gilts and sows are grouped under the term SMEDI, which stands for stillbirths, mummies, embryonic death, and infertility [3,4]. The primary (putative) viral agents suspected to cause SMEDI include porcine circovirus type 2 (PCV2), porcine parvovirus 1 (PPV1), and porcine reproductive and respiratory syndrome virus (PRRSV) [4,5,6]. Recent advances in detection and sequencing technologies have identified emerging viruses in samples from sows affected by PRF. These emerging viruses include porcine circovirus type 3 (PCV3) [7,8,9] and new species of porcine parvoviruses (PPV2 to PPV7) [10], which are collectively referred to as novel porcine parvoviruses (nPPVs) [11]. Two key findings support the classification of these emerging pathogens as potential causes of PRF: (i) they are frequently detected in cases of SMEDI, and (ii) they often occur alongside primary PRF viruses, indicating instances of coinfection.

Coinfections involving PCV2/PRRSV [12,13] and PCV2/PPV1 [14,15] have historically been the most common in cases of PRF-associated SMEDI. However, recent literature has identified other coinfections, such as PCV2/PCV3 and various strains of nPPVs, in concurrence with these two PCVs [10,16]. Viral coinfections can lead to several outcomes: (i) viral interference, where one virus inhibits the replication of another; (ii) increased replication of one of the coinfecting viruses, which may result in more severe clinical signs; and (iii) the coexistence of concurrent viruses without significant changes in their individual replication rates [17]. When it comes to PRF, the effects of viral coinfections are not yet fully understood. This highlights the need for further research to clarify the clinical signs, the microscopic and macroscopic lesions in both fetal and maternal tissues, the impact on viral loads of the concurrent viruses, and how these coinfections affect reproductive parameters.

This study investigated the presence of primary viruses associated with PRF, including PCV2, PPV1, and PRRSV, as well as emerging viruses such as PCV3, PCV4, and nPPV types PPV2 to PPV8. The research focused on two groups of sows: primiparous (gilts) and multiparous sows. To achieve this, a longitudinal follow-up study was conducted involving these two groups of sows and their offspring during three phases: pregnancy, farrowing, and lactation. Additionally, the effects of viral coinfections on PRF were evaluated by analyzing viral loads and histopathological lesions in tissue samples from both maternal and fetal sources.

## 2. Materials and Methods

### 2.1. Herd Selection and Sample Collection

The study was conducted from May 2022 to April 2023 in four herds (H1 to H4) of closed-cycle pigs across the three Colombian provinces with the highest pig production: Antioquia, Cundinamarca, and Atlántico. The herds were selected based on the prior detection of at least one of the primary PRF viruses (PCV2, PPV1, or PRRSV) in the sow population. Table S1 provides an overview of the four evaluated herds, including their geographic location, sow inventories, vaccination protocols, reproductive parameters, and the samples collected for both gilts and sows during the longitudinal monitoring. The reproductive parameters assessed included the total born piglets index, the piglets born alive index, the percentage of mummies, the percentage of stillborn piglets, and the percentage of preweaning mortality (PWM). Sows, both primiparous and multiparous, from all four herds were vaccinated against PCV2 and PPV1 according to the routine protocols established by each herd and following the recommendations from the vaccine manufacturers. The commercial names of the PCV2 and PPV1 vaccines, along with the vaccination ages of the gilts on each herd, are detailed in Table S1. It is important to note that there is currently no vaccination against PRRSV available in Colombia, and no vaccines are on the market for this virus.

**Figure 1:**
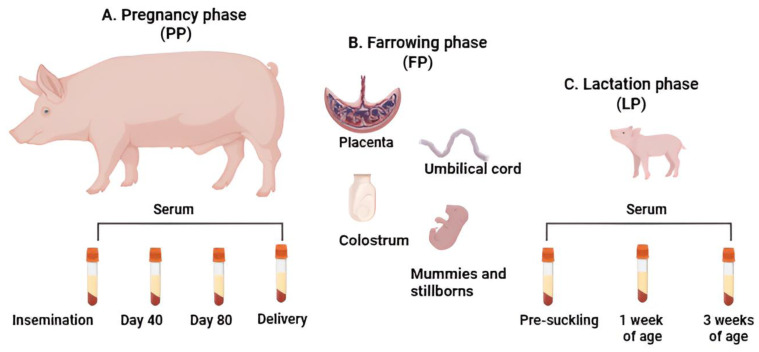
Schematic representation of this study involved longitudinal monitoring of gilts (primiparous sows, PS) and sows (multiparous sows, MS) to detect viruses associated with SMEDI during three distinct phases. (**A**). Pregnancy phase (PP): Blood samples were collected every 40 days, from insemination until delivery. A total of four samples were collected from the same PS (*n* = 5) and MS (*n* = 5) across each of the four herds (*n* = 4 herds). (**B**). Farrowing phase (FP): Blood and tissue samples were collected from the PS, MS, and their newborn piglets (*n* = 36), along with tissues from any released mummies and stillbirths. (**C**). Lactation phase (LP): Piglets were monitored longitudinally for three weeks, with blood samples collected before suckling and at weeks 1 and 3 of age (*n* = 36). The figure was created using Biorender.com.

In each of the four chosen herds, we randomly chose 10 clinically healthy animals. The original plan was to select five gilts, known as primiparous sows (PSs), and five sows, referred to as multiparous sows (MSs). However, we could not achieve this distribution due to confusion during the selection process. Ultimately, we followed 18 PS and 22 MS. To ensure traceability, each animal was ear-tagged and followed longitudinally through three phases: (i) the pregnancy phase (PP), (ii) the farrowing phase (FP), and (iii) the lactation phase (LP). During PP, we collected blood samples via jugular venipuncture from each group of ten animals. Sampling began on the day of insemination and continued at 40-day intervals until delivery, resulting in four blood samples per PS and MS (totaling 40 blood samples per herd). In the FP, the delivery of the PS and MS was supervised obstetrically. We collected colostrum, placenta, and umbilical cord samples during this phase. Additionally, pre-suckling blood samples were collected from the umbilical cords of nine newborn piglets from each litter. Following the piglets’ birth, we continued monitoring them during LP. We collected blood samples at three time points: pre-suckling (from the umbilical cord, as previously described) and via venipuncture at weeks 1 and 3 of age. The serum samples from each time point were processed in pools of three sera, resulting in three serum samples per litter for each time point (totaling 9 samples per herd and 36 for the 4 herds). The samples were collected and immediately transported to the animal virology laboratory at the Facultad de Medicina Veterinaria y de Zootecnia, Universidad Nacional de Colombia, Sede Bogotá. Blood samples were centrifuged at 1000× *g* (Sorvall ST 8^®^, Langenselbold, Germany) for 10 min. The serum was then stored at −80 °C until further processing. At the FP, tissue samples (spleen, kidney, brain, heart, lung, liver, and lymph node) were collected from mummies larger than 17 cm or from stillbirths. For mummies less than 17 cm in length, thoracic macerate was collected. Two portions of each tissue, approximately 5 g each, were retrieved. One portion was refrigerated and sent to the laboratory for storage at −80 °C until further molecular analysis. The second portion was fixed in 10% neutral buffered formalin for histopathological studies performed using hematoxylin–eosin staining. Figure 1 schematically illustrates the experimental design of this study, distinguishing the samples collected during the three evaluated phases.

### 2.2. Serology

Serum samples collected during the three phases of the study were evaluated via ELISA to determine antibody (Ab) titers against various viruses, including PRRSV, PCV2, PPV1, and PCV3. According to the manufacturers’ instructions, anti-PRRSV Abs were detected using the IDEXX PRRS X3 Ab Test^®^ kit (IDEXX, Westbrook, ME, USA), with positive sera defining as having S/P values of ≥0.4 (cut-off). For PCV2, the INgezim Circovirus IgG^®^ kit (Ingenasa, Madrid, Spain) was utilized, while the INgezim PPV COMPAC^®^ kit (Ingenasa, Madrid, Spain) was used for PPV1, with positive samples showing S/P values of ≥0.3 (cut-off). Anti-PCV3 Abs were assessed at two specific time points: in PS and MS at delivery and in piglets before suckling, using the AsurDx TM Porcine circovirus 3 antibodies Test^®^ kit (BIOSTONE, Southlake, TX, USA), where a positive S/P value was considered to be ≥0.5 (cut-off).

### 2.3. Processing Samples and Detecting Viruses

As previously mentioned, serum and tissue samples were stored at −80 °C. They were thawed at room temperature to prepare the tissue samples for analysis. A portion of 0.5 g from each sample was resuspended in 5 mL of 10% phosphate-buffered saline (PBS). Each sample was then homogenized using a handheld homogenizer (Fisherbrand^®^ 150, Thermo Fisher Scientific, PA, USA). After homogenization, the mixture was centrifuged for 15 min at 2000× *g* at 4 °C, and 200 µL of the supernatant was collected for nucleic acid extraction. Colostrum samples (5 mL) were centrifuged at 2000× *g* for 15 min at 4 °C, and the fat layer was removed. From this, 200 µL of the liquid phase was collected for nucleic acid extraction. Following the manufacturers’ instructions, nucleic acids were extracted from 200 µL of serum, colostrum, and tissue supernatant using the High Pure Viral Nucleic Acid kit^®^ (Roche, Mannheim, Germany). All extracted samples were eluted in 100 µL of elution buffer. Each extraction was divided into two aliquots: one for detecting DNA viruses and another for detecting PRRSV. The aliquots were stored at −80 °C until further processing. Additionally, each sample was tested for the porcine β-actin gene to confirm DNA integrity, using primers previously reported [18]. Complementary DNA (cDNA) synthesis was performed using the High-Capacity RNA-to-cDNA^®^ Kit (Thermo Scientific, Carlsbad, CA, USA) following the manufacturers’ protocol to detect PRRSV. The reaction utilized 10 µL of RNA, resulting in a final volume of 20 µL. Various viruses were detected using three PCR methods: (i) real-time PCR with TaqMan^®^ probes (Macrogen Inc, Seoul, South Korea), this method was used to detect PCV2, PCV3, PPV1, and PRRSV; (ii) real-time PCR with SYBR Green chemistry—this method was employed for detecting PPV2 through PPV7; (iii) end-point PCR was used for detecting PCV4 and PPV8. The specific primers and probes for each target virus are listed in Table S2, which includes the sequences and original references for each set of primers and probes. 

Real-time PCR reactions for PCV2, PCV3, PPV1, and PRRSV were conducted using protocols adapted from [19,20,21,22]. Each reaction was performed in a total volume of 20 µL, which included 400 nM of each primer, 250 nM of each probe, 2 µL of template DNA/cDNA, and 10 µL of LightCycler^®^ 480 Probes Master Mix (Roche, Mannheim Germany). The thermal cycling conditions began with an initial activation at 95 °C for 10 min, followed by 45 cycles consisting of denaturation at 95 °C for 15 s, annealing at 60 °C for 45 s, and extension at 72 °C for 2 s. For SYBR Green-based real-time PCR, targeting PPV2 to PPV7, this was conducted using primers and protocols adapted from [23,24,25,26,27]. These reactions were performed in a total volume of 20 µL, utilizing 10 µL of SsoAdvanced™ Universal SYBR^®^ Green Supermix (Bio-Rad, Hercules, CA, USA), with a final primer concentration of 400 nM, and 2 µL of template DNA. The amplification protocol started with an initial activation at 95 °C for 10 min, followed by 45 cycles of 95 °C for 1 min, 60 °C for 24 s, and 72 °C for 2 s. Additionally, a melting curve analysis was conducted from 65 °C to 95 °C in 0.5 °C increments (5 s per step) to verify the specificity of the amplified products. All real-time PCR reactions using TaqMan^®^ or SYBR Green detection chemistry were performed in duplicate in the LightCycler^®^ 480 system (Roche, Burgess Hill, UK). End-point PCRs for PPV8 and PCV4 were conducted using protocols adapted from [28,29]. The reactions were carried out in 25 µL of reaction volumes, which included 0.25 U of GoTaq^®^ Flexi DNA Polymerase (Promega, Madison, WI, USA), 1X Taq reaction buffer, 2 mM MgCl_2_, 200 µM of each dTNP, 400 nM of each primer, and 2 µL of extracted DNA. The thermal cycling protocol comprised an initial denaturation step at 94 °C for 5 min, followed by 35 cycles of 94 °C for 30 s, 58 °C for 30 s, and 72 °C for 30 s, with a final extension step at 72 °C for 10 min. PCR products were analyzed by electrophoresis on 1.5% agarose gels, which were stained with VWR^®^ EZ-Vision^®^ Dye (VWR, Radnor, PA, USA) and visualized under UV light.

Field samples (tissues) previously diagnosed as positive through conventional PCR were utilized to create positive controls and standardize real-time PCR for each virus. These amplicons were cloned using the TOPO TA^®^ cloning kit (Invitrogen, Carlsbad, CA, USA) and transformed into One Shot^®^ Chemically Competent *E. coli* (Invitrogen, Carlsbad, CA, USA). The presence of the insert and its correct orientation were confirmed through sequencing conducted by SSiGMol (Servicio de Secuenciación y Análisis Molecular, Instituto de Genética, Universidad Nacional de Colombia, Bogotá). The recombinant plasmids were then purified using the plasmid maxi kit^®^ (Qiagen, Hilden, Germany) and quantified using OD260 on a Nano200^®^ spectrophotometer (Thermo Scientific, Wilmington, DE, USA). The limit of detection (LoD) for qPCR was established using two complementary methods: (i) analytical LoD—serial 10-fold dilutions (ranging from 10^1^ to 10^9^ copies/reaction) of each recombinant plasmid were prepared in nuclease-free water to create standard curves. Each dilution was tested in triplicate across three independent runs. (ii) Matrix LoD—negative biological matrices (including serum, colostrum, and tissue homogenates from negative pigs) were spiked with decreasing concentrations (10^5^ to 10^0^ copies/reaction) of viral targets. Following WOAH guidelines, this approach determined the matrix-specific LoD with a 95% detection probability. Three replicates were performed for each dilution. The conversion to DNA copy number was calculated using the formula number of copies = [ng × (6.022 × 10^23^)]/[length × (1 × 10^9^) x 650]. The qPCR efficiency (E) was evaluated through the formula E = 10(−1/slope) − 1. The slope was determined using linear regression between the crossing points (Cq) and corresponding log-transformed viral copy numbers. The R2 (coefficient of determination) was calculated to assess the goodness of fit of the regression line fit in explaining the relationship between dilution and Cq, with values > 0.98 considered acceptable. Intra-laboratory repeatability was assessed by selecting three dilutions representing high (10^6^ copies/reaction), medium (10^4^ copies/reaction), and low (10^2^ copies/reaction) viral concentrations. Two operators tested each dilution on two separate days. PCR performance metrics are detailed in Table S3. The analytical specificity of each assay was evaluated using a panel of various swine DNA pathogens, including PCV2, PCV3, PPV1 to PPV7, and *Mycoplasma hyopneumoniae*.

Based on our validation studies, we established positivity thresholds for TaqMan^®^ probe-based assays for PCV2, PCV3, PPV1, and PRRSV at a Ct value ≤ 37. For SYBR Green-based assays for PPV2 through PPV7, the threshold was set at a Ct value ≤ 35, provided there was an appropriate melting curve. Results were reported as log_10_ viral DNA/RNA copies/mL for serum and colostrum samples, or copies/g for tissue samples. We compared viral loads between viruses when the same detection method was used, specifically for PCV2, PCV3, and PPV1, all detected using probe-based qPCR. Comparisons were not made between the probe-based qPCR targets and those detected with SYBR Green (such as nPPVs) due to differences in detection chemistry and performance parameters.

### 2.4. Sequencing of Identified Viruses and Phylogenetic Analysis

The nucleotide (nt) coding region sequencing of PCV2 and PCV3-ORF2 was conducted using specific primers as previously reported [30,31]. For PRRSV-ORF5, sequencing was performed using primers described in earlier studies [32]. It is important to note that only samples with a Ct < 30 in qPCR could be sequenced. Sequencing for PCV4, PPV8 (which was not detected), and PPVs (PPV1 through PPV7) was not conducted mainly due to negative results or insufficient viral loads. PCR assays for sequencing were carried out in a total volume of 25 μL, which included 1 U of AccuPrime^®^ Taq DNA Polymerase (Invitrogen, Waltham, MA, USA), 1X AccuPrime PCR Buffer I, 400 nM of each primer, and approximately 100 ng of extracted DNA. The PCR conditions were as follows: an initial denaturation at 94 °C for 1 min, followed by 35 cycles consisting of denaturation at 94 °C for 30 s, annealing at 57 °C for 30 s, and extension at 68 °C for 1 min. After amplification, the PCR products were purified using the QIAquick PCR purification kit^®^ (Qiagen, Hilden, Germany), according to the manufacturers’ instructions. Finally, bidirectional Sanger sequencing was conducted by SSiGMol (Servicio de Secuenciación y Análisis Molecular, Instituto de Genética, Universidad Nacional de Colombia, Bogotá).

For PCV2, four complete ORF2 nt sequences were obtained and deposited in GenBank database under the following accession numbers: PV640824 to PV640827. These sequences were aligned with 52 reference sequences selected based on two criteria: (i) previously reported Colombian strains and (ii) representative sequences from all established PCV2 genotypes, ranging from PCV2a to PCV2h. For PCV3, one complete ORF2 nt sequence was obtained (GenBank accession number PV640828) and was compared to 45 reference sequences from the GenBank database. This comparison included representatives from established clades (PCV3a and PCV3b) and previously reported Colombian strains. For PRRSV, one ORF5 nt sequence was obtained (GenBank accession number PV640829) and compared with 65 reference sequences, which included previously reported Colombian strains and representatives from all nine established lineages of PRRSV-2 (L1 through L9).

Multiple sequence alignments were conducted using the ClustalW algorithm, part of the MEGA 7.0 software [33]. Prior to phylogenetic analysis, the best-fit model for nt substitution was determined for each viral dataset using the Bayesian Information Criterion (BIC) with the model selection tool in MEGA 7.0. For both the PCV2 and PCV3 datasets, the Tamura-Nei model with a discrete Gamma distribution (TN93 + G) was identified as the optimal evolutionary model. Conversely, the Kimura 2-parameter model with a discrete Gamma distribution and a proportion of invariable sites (K2 + G + I) was selected for the PRRSV dataset. Phylogenetic trees were constructed using the Maximum Likelihood (ML) method implemented in MEGA 7.0 with the respective models chosen. The robustness of these phylogenetic trees was evaluated through bootstrapping with 1.000 replicates.

### 2.5. Histopathology

For histopathological evaluation, tissue samples from the placenta and umbilical cord of PS and MS, as well as from the fetuses (including lung, heart, liver, spleen, kidney, and brain), were fixed in 10% buffered formalin and embedded in paraffin. The sections were then stained with hematoxylin and eosin for examination under light microscopy. This procedure adhered to the standardized protocol established at the veterinary pathology laboratory at the Facultad de Medicina Veterinaria y de Zootecnia, Universidad Nacional de Colombia, Sede Bogotá.

### 2.6. Statistical Analysis

The normality of data distribution was assessed using the Shapiro–Wilk test. Following this, several statistical analyses were performed: (i) the nonparametric Mann–Whitney U test was employed to compare reproductive parameters, including percentage of mummies, percentage of stillbirths, total born piglets, and piglets born alive between herds as well as between PS and MS groups. (ii) The nonparametric Wilcoxon test was used to compare Ab titers and viral loads among different herds, between PS and MS groups, and across various sampling time points. (iii) The Spearman correlation test was calculated to determine the association between the presence of the virus at delivery and the viral load detected in the placenta, umbilical cords, colostrum, and pre-suckling serum. This test also assessed correlations in Ab titers between the PS and MS groups at delivery and during lactation. (iv) The nonparametric Kruskal–Wallis test was conducted to compare viral loads associated with different types of infection (mono-infections and coinfections) in PP, FP, and LP phases. (v) A binomial logistic regression analysis evaluated the relationship between the dependent variable (virus-positive fetus) and several independent variables, including PS or MS, the type of infection, and the vaccination status against PCV2 and PPV1. It is important to note that the association analyses between viral loads of PCV2, PCV3, PPV1, and PRRSV with the nPPVs (PPV2 to PPV7) were not performed due to differences in the methods used for quantification. All statistical analyses were conducted using the “PCR” package in the RStudio program version 4.3.0 [34]. Statistical significance was set at *p* < 0.05 for all tests.

## 3. Results

### 3.1. Reproductive Parameters

This study followed the entire population of sows (both PS and MS) longitudinally throughout the PP, FP, and LP phases. No clinical signs of disease were observed during any of the three phases of the study. However, notable differences in reproductive parameters were observed among three herds (H1, H2, and H3) compared to herd H4. The number of piglets born alive varied, with H1, H2, and H3 showing rates between 12% and 13.3%, while H4 had a higher rate of 16%. Regarding mummified fetuses, H1 had a rate of 20%, in contrast to just 4.3% in H4. The percentage of stillborn pigs ranged from 8.8% to 10.1% in H1, H2, and H3, whereas H4 had a lower percentage of 3.6%. Finally, the PWM was 7.6% in H2, 4.1% in H1, and 0.7% in H4. Table S1 outlines the reproductive parameters recorded for each herd during the study period. The survival history of the study animals is as follows: At the beginning of the study, there were a total of 40 animals, consisting of 18 PSs and 22 MSs. After 80 days of pregnancy (during PP), 2 MSs from the H4 group died, despite no record of illness, resulting in a total of 38 animals at the time of delivery (18 PSs and 20 MSs). During the delivery process, serum samples were collected from all 38 animals. Unfortunately, two additional animals from the H3 group, one from the PS group and one from the MS group, died during this period. Samples of umbilical cord and colostrum were collected from one of the deceased animals, resulting in 37 animals remaining in FP for analyses in this phase. For the LP phase, 36 animals were available (17 PSs and 19 MSs), which led to 36 litters being monitored. A total of 602 releases (fetuses and neonates) were reported for these 36 litters. In terms of mortality, there were 49 stillborns (8.3%) and 58 mummified fetuses (9.6%). No statistically significant differences were found between PS and MS when comparing the number of stillborns (*p* = 0.23), mummy fetuses (*p* = 0.37), and live-born piglets (*p* = 0.25) (Figure S1A). However, in H4, significantly more live-born piglets (*p* = 0.04) were observed in the MS group compared to the PS group (Figure S1B).

### 3.2. Viremia and Serological Assessments in Both Primiparous and Multiparous Sows Throughout the Pregnancy Phase

We detected viral circulation in all four herds during the PP. Specifically, PCV2, PCV3, and PPV1-DNA were found in both PS and MS across all four herds (H1 to H4). In contrast, PRRSV-RNA was identified exclusively in PS and MS of H1 from day 40 of pregnancy until farrowing. Additionally, Abs against PRRSV were detected in herds H1 and H3. Regarding nPPVs (PPV2 to PPV8), PPV3 was identified in four sows (three PS and one MS) in herds H1 and H4, detected at insemination and on day 80 of pregnancy, respectively. Both PPV4 and PPV6 were simultaneously detected on day 40 of pregnancy in one MS from herd H1, while PPV6 was found in two MSs from herd H3 on day 80 of pregnancy. Notably, neither PCV4 nor PPV8 were detected in any of the four herds during the PP period. Table 1 and Table S4 and Figures S2 and S3 provide detailed information on Ab titers and viral loads for each virus, comparing the PS and MS across the herds during PP.

The findings regarding PCV2 in PS and MS during the PP indicated that 35% (14/40) of the sows (6 PSs and 8 MSs) were viremic at the time of insemination. This proportion increased, reaching 86.8% (33/38) at delivery (15 PSs and 18 MSs). Notably, 17 sows (8 PSs and 9 MSs) maintained a continuous viremia for more than 40 days, although the PCV2 viral loads consistently remained below 4.2 Log10 copies (Ct > 30), suggesting a subclinical infection in the PCV2-positive sows. Regarding anti-PCV2 Abs, all PSs and MSs displayed detectable levels during PP (S/P value > 0.5). Statistical analysis revealed no significant differences in Ab titers (*p* = 0.91) or viral loads (*p* = 0.85) for PCV2 when comparing PS and MS across all herds. Turning PCV3, 37.5% of the sows (15/40) (7 PSs and 8 MSs) were viremic at insemination. This proportion decreased to 13.1% (5/38) at delivery, with no significant differences (*p* = 0.1) observed between viremic PSs and MSs. Throughout PP, all PCV3-positive sows remained viremic for more than 40 days, and the PCV3 viral loads were below 4 Log10 copies (Ct > 30.5), indicating a subclinical infection. Moreover, no significant differences in PCV3 viral load (*p* = 0.76) were identified between PS and MS. Anti-PCV3 Abs were detected in 57% (22/38) of the evaluated samples, showing significant differences (*p* = 0.045) between PSs and MSs at delivery, with MSs exhibiting the higher titers (Table 1). Regarding PPV1, 47.5% (19/40) of sows (10 PSs and 9 MSs) were found to be viremic at the time of insemination. This percentage decreased to 34.2% (13/38) at delivery, comprising 7 PSs and 6 MSs. Seven sows (four PSs and three MSs) maintained continuous viremia for over 40 days. Throughout the PP, the viral loads of PPV1 remained below 3.9 log10 copies (Ct > 28). In contrast, anti-PPV1 Abs were detected in 65% (26/40) of sows at insemination, with no significant difference between PS and MS (*p* = 0.09). However, the titers of anti-PPV1 Abs decreased over time; by delivery, they were present in 57.9% (22/38) of sows, with significantly higher titers observed in MS (*p* = 0.04) (Table 1). For PRRSV, all sows tested were negative at insemination. However, by day 40 of pregnancy, 20% (8/40) of sows (4s PS and 4 MSs) in H1 developed viremia. The viral loads in all PRRSV-positive sows remained below 3.1 Log10 copies (Ct > 35). Regarding anti-PRRSV Abs, 25% (10/40) of the evaluated sows (5 PSs and 5 MSs), all from H3, were seropositive at insemination. This seropositivity increased to 34.2% (13/38) by delivery, including sows from H1. Intra-herd analysis revealed highly significant differences (*p* < 0.001) in anti-PRRSV Abs titers in H1 between sampling time points; this was attributed to the 100% seropositivity detected at delivery in both PS and MS. As previously stated in the materials and methods, it is important to note that no vaccination against PRRSV is currently available in Colombia, and no vaccines are available on the market. Therefore, detecting either PRRSV-RNA or anti-PRRSV Abs indicates infection with a wild-type PRRSV strain.

#### Coinfections Caused by Viruses During the Pregnancy Phase

**Figure 2:**
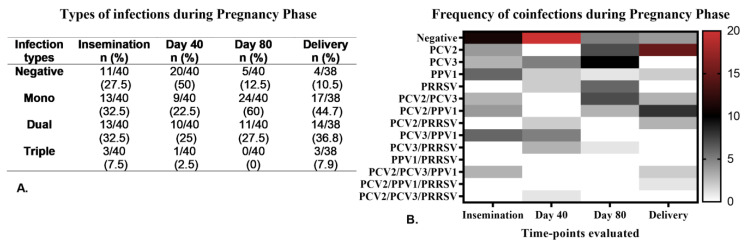
Types of viral infection (mono- and coinfections) detected in the serum of sows (primiparous and multiparous) from the four herds evaluated throughout the pregnancy phase (PP). (**A**). Frequency of infection types (mono, dual, and triple) at the four sampling times during PP. (**B**). A Heat map illustrating the frequency of mono- and coinfections during PP. The color scale (0–20) represents the frequency of infection, where red indicates the highest frequency, black denotes moderate frequency, and white signifies no infection.

During the PP, we observed no differences in the types of infections (mono- and coinfections) between the sows in the study groups (PS and MS). As a result, we analyzed the coinfections across the entire population of sows included in the study. Figure 2A shows the distribution of infection types (mono- and coinfections) across the four herds during the PP, where both mono- and dual infections were frequent, each exceeding 22%. The percentage of sows negative for all tested viruses was highest on day 40 of pregnancy and lowest at delivery time. Among the mono-infections identified at the four sampling points, PPV1 had the highest frequence at insemination, PCV3 was most common on days 40 and 80 of pregnancy, and PCV2 was most frequent at delivery (Figure 2B). In terms of dual coinfections, PCV3/PPV1 was most frequently observed at insemination and on day 40, while PCV2/PCV3 was most common on day 80, and PCV2/PPV1 was predominant at delivery. Triple coinfections were rare during PP, with PCV2/PCV3/PPV1 being the most frequently encountered (Figure 2B).

**Figure 3:**
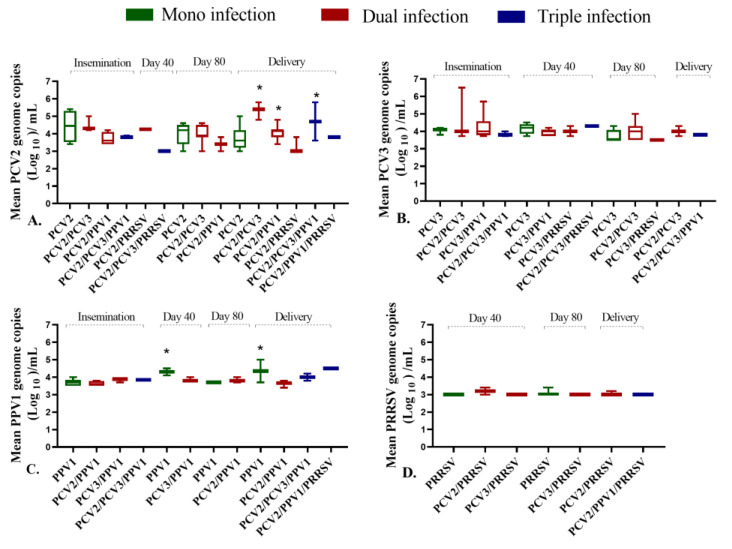
Association between viral load and type of infection during pregnancy phase (PP). This box plot illustrates the viral load of PCV2, PCV3, PPV1, and PRRSV according to the type of infection: green represents mono-infection, red indicates dual infection, and blue signifies triple infection. (**A**). PCV2 viral load; (**B**). PCV3 viral load; (**C**). PPV1 viral load; (**D**). PRRSV viral load. * Significant differences were observed (*p* < 0.05).

During PP, coinfections involving PCV2 affected the viral load of this virus. Sows that had coinfections with PCV2/PCV3, PCV2/PPV1, and PCV2/PCV3/PPV1 showed significantly higher (*p* = 0.04) PCV2 viral loads at the time of delivery (*p* = 0.04) compared to those where PCV2 was detected in mono-infection or PCV2/PRRSV coinfection (Figure 3A). In contrast, the viral load of PPV1 was not influenced by coinfections; it was significantly higher (*p* = 0.04) in sows with PPV1 mono-infection on day 40 of pregnancy and at the time of delivery (Figure 3C). No significant differences were found in the viral loads for PCV3 or PRRSV based on the type of infection (Figure 3B,D). Detailed viral load results for each type of infection are exhibited in Table S5.

**Figure 4:**
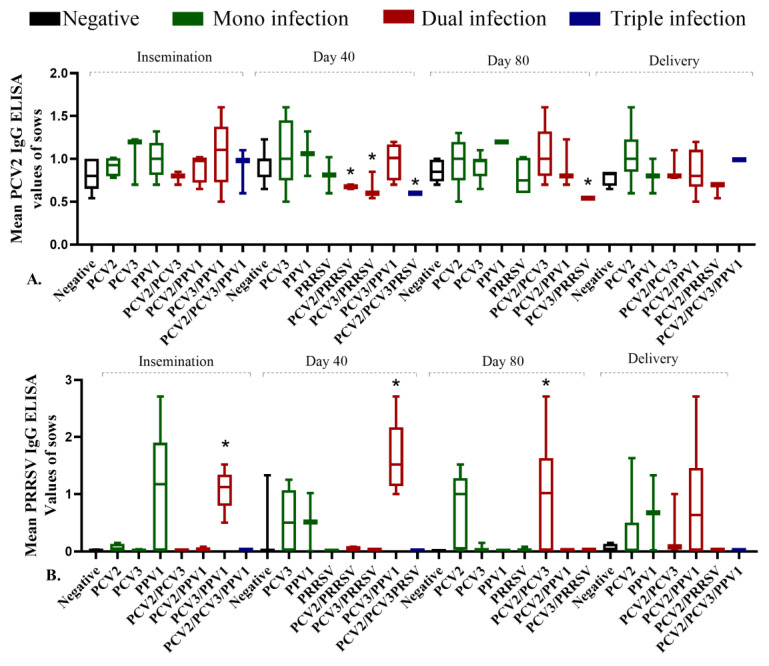
Relationship between antibody (Ab) titers for anti-PCV2 and anti-PRRSV and the type of infection detected throughout pregnancy phase (PP) in the evaluated sow population. (**A**). Anti-PCV2 Ab titers, (**B**). Anti-PRRSV Ab titers. * Significant differences (*p* < 0.05). The colors represent different infection types: Green indicates mono-infection, red indicates dual coinfection, and blue indicates triple coinfection.

Analysis of Ab responses related to the type of infection revealed significant associations. At day 40 of pregnancy, anti-PCV2 Ab titers were significantly lower (*p* = 0.04) in all sows (both PS and MS) where PRRSV-associated coinfections were detected (PCV2/PRRSV and PCV3/PRRSV), compared to other types of coinfections (*p* = 0.34). This effect persisted through day 80 of pregnancy for the PCV3/PRRSV coinfection (*p* = 0.04) (Figure 4A). Conversely, significantly higher anti-PRRSV Ab titers were observed in sows with PCV3/PPV1 or PCV2/PCV3 coinfections (*p* = 0.05). This was particularly notable for PCV3/PPV1 at insemination and on day 40 of pregnancy and PCV2/PCV3 on day 80 (Figure 4B). We did not find any significant differences in anti-PPV1 Ab titers on the type of infection during the PP. 

### 3.3. Viral Detection in Primiparous and Multiparous Sows During the Farrowing Phase

During FP, we concentrated on vertical viral transmission by analyzing samples of umbilical cord and colostrum from 37 PS and MS. We also collected tissue and serum samples from their offspring, which included mummified fetuses, stillborns, and newborn piglets. Viral genomes of PCV2, PCV3, and PPV1 were identified in all herds (H1 to H4), while PRRSV-RNA was detected only in herd H1. During this phase, PCV4 and nPPVS (PPV2 to PPV8) were not detected. The viral frequency in placenta samples (*n* = 37) was as follows: PCV2 72.98% (27/37), PPV1 43.25% (16/37), PCV3 21.63% (8/37), and PRRSV 13.52% (5/37). For umbilical cord samples (*n* = 37), the frequency was PCV2 86.49% (32/37), PPV1 40.54% (15/37), PCV3 24.33% (9/37), and PRRSV 10.81% (4/37). For colostrum samples (*n* = 37), the data were PCV2 72.98% (27/37), PPV1 35.14% (13/37), PCV3 29.73% (11/37), and PRRSV 8.11% (3/37). Table 2 displays the frequencies and viral loads for the viruses examined in the placenta, umbilical cord, and colostrum samples from PS and MS during FP. The detected viral loads were as follows: PCV2 (3.9 to 4.2 log10 copies/g or mL), PPV1 (3.8 to 4.1 log10 copies/g or mL), PCV3 (3.7 to 4.0 log10 copies/g or mL) and PRRSV (3 to 3.4 log10 copies/g or mL). No significant differences (*p* > 0.05) were observed between PS and MS in the viral loads for these viruses across the three types of samples evaluated (Table 2). The detailed frequencies and viral loads for each herd are represented in Table S6.

**Figure 5:**
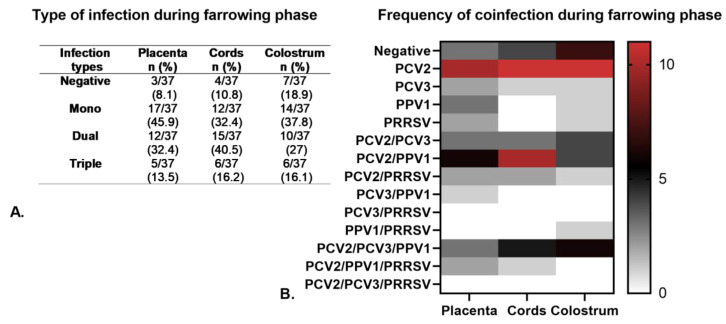
Types of viral infection (mono- and coinfections) detected in placenta, umbilical cords, and colostrum from sows (both primiparous and multiparous) across the four herds evaluated during the farrowing phase (FF). (**A**). Frequency of different types of infection (mono, dual and triple) detected during FP. (**B**). A heat map depicting the frequency and types of infection identified during FP. The color scale ranges from 0 to 20, where red represents the highest frequency of infection, black indicates a moderate frequency, and white signifies no infection.

Regarding the type of infection, mono-infections were the most common during FP, comprising 32.4 to 45.9% of cases, with PCV2 as the most frequent virus. The frequency of dual coinfections ranged from 27 to 40.5%, with PCV2/PPV1 being the most frequently observed. Triple coinfections occurred in 13.5 to 16.1% of cases, with the most common combination being PCV2/PCV3/PPV1 (Figure 5A,B). Importantly, no significant association (*p* > 0.05) was found between dual or triple coinfections and the viral load of any specific virus when compared to mono-infection.

**Figure 6:**
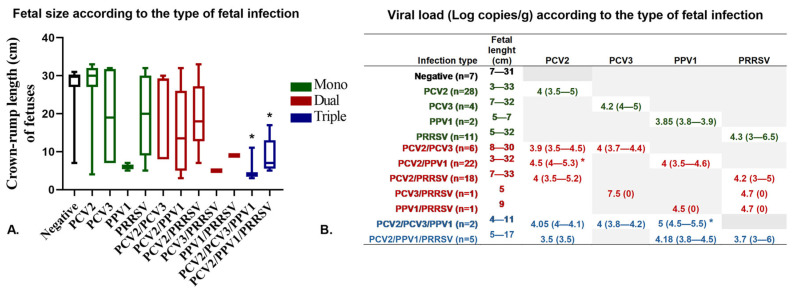
Association between infection types (mono- and coinfections), viral loads, and fetal crown-rump length (CRL). (**A**). This box plot illustrates the relationship between the type of infection (mono- and coinfections) detected in fetuses (stillbirths and mummies) and fetal CRL. (**B**). The association between fetal CRL and viral load (log copies/g) indicates the mean viral load and the minimum and maximum values for each type of infection. Mono-infections are represented in green, dual infections in red, and triple infections in blue. * Significant differences are indicated (*p* < 0.05).

We examined the offspring of the evaluated sows and observed a total of 602 piglets (both living and dead) across the four sampled herds. Among these, 107 were classified as fetuses, which included 49 stillborns (8.3%) and 58 mummies (9.6%). Our viral detection and quantification results revealed a high frequency of PCV2 in the fetuses, with a positivity rate of 75% (81/107). Specifically, PCV2 was found in mummies with crown-rump length (CRL) ranging from 4 to 30 cm, with the mean viral load varying from 3.5 to 4.5 log10 copies/g (Figure 6). The rate of PCV3 positivity in fetuses was 13% (14/107), and its mean viral load ranged from 4 to 7.5 log10 copies/g. Notably, one fetus (with a CRL < 17 cm) exhibited an unusually high PCV3 viral load of 7.5 log10 copies/g (Ct value 18), marking the highest viral load detected in this study. This particular fetus also showed a PCV3/PRRSV coinfection (Figure 6B). As for PPV1, the positivity rate in fetuses was 30.85% (33/107), with a mean viral load ranging from 3.85 to 5 log10 copies/g (Ct > 27). We identified PPV1 mono-infections in mummies with CRL ranging from 4 to 10 cm and detected coinfections in those with CRL between 4 and 32 cm (Figure 6). Finally, PRRSV was found only in herd H1, where it was identified as a mono-infection in 33.65% (36/107) of fetuses, with CRL ranging from 5 to 33 cm, showing a mean viral load between 3.7 and 4.7 log10 copies/g (Ct > 29). Additionally, we made two noteworthy observations regarding coinfections in FP: (i) we detected the PCV3/PRRSV and PPV1/PRRSV coinfections in mummies with CRL < 17 cm, and (ii) the triple coinfection of PCV2/PPV1/PRRSV was found in mummies with CRL < 17 cm, which was statistically significant (*p* = 0.045) compared to other types of coinfections (Figure 6A). Among the 107 stillbirths and mummies evaluated, only 6.55% (7/107) tested negative for all viruses. Mono-infections accounted for 42.06% (45/107), with PCV2 being the most common. Dual coinfections comprised 44.86% (48/107), with PCV2/PPV1 being the most frequently observed. Triple coinfections constituted 6.55% (7/107), with PCV2/PPV1/PRRSV being the predominant combination. Detailed information regarding the rates and types of infections (mono- and coinfections) in mummies and stillbirths can be found in Table S7, while Table S8 and Figure S4 present the frequency of PRRSV, PCV2, PCV3, and PPV1 across herds.

We found a significant association between PCV2 viral load and coinfections during the FP. Specifically, the PCV2 viral load was significantly higher in cases of PCV2/PCV1 coinfection (*p* < 0.01) compared to PCV2 mono-infection (Figure S5A). Additionally, the viral load of PPV1 was significantly elevated (*p* < 0.01) in fetuses where a triple coinfection of PCV2/PCV3/PPV1 was detected, relative to PPV1 mono-infection (Figure S5C). For PCV3, although we identified a difference in viral load in PCV3/PRRSV coinfection cases, we could not determine the statistical significance due to the presence of only a single sample. There were no significant differences in viral loads among the other coinfections or for the individual viruses (Figure S5B,D). Table S9 summarizes the mean viral loads (log10 copies/g) for each virus across the fetuses’ different infection types (mono-infections and coinfections). No significant differences were observed in viral loads or the type of infection between fetuses from PS and MS groups.

We conducted a multivariate analysis to determine the probability of detecting each virus in fetuses (the dependent variable) based on herd-specific or fetal-specific factors. Independent variables included whether the fetuses tested positive or negative for PPV1, PCV2, PCV3, and PRRSV, as well as the vaccination status of PS or MS against PCV2 and PPV1. The analysis provided two significant findings: First, there was a notable increase (*p* < 0.01) in the probability of detecting PPV1-positive fetuses in PCV2/PPV1 coinfection cases. Second, PRRSV-positive fetuses were more commonly found in those who tested negative for the other viruses. Additionally, no relationship was observed between parity status (PS or MS) and the vaccination status for PCV2 or PPV1. The results of the multivariate analysis are presented in Table S10.

### 3.4. Detection of Viruses and Antibodies in Piglets During the Lactation Phase

During LP, we detected several viruses in all four herds, including PCV2-, PCV3-, PPV1-DNA, and Abs against PPV1. Notably, Abs against PCV3 were only found in pre-suckling pigs from two herds (H2 and H3). PRRSV-RNA was exclusively identified in piglets from herd H1, while Abs against PRRSV was present in both herds H1 and H3. Regarding nPPVs, DNA for PPV2 and PPV6 was found in H1 and H2, with each virus occurring in 2.77% of litters (1/36) at week 3. Additionally, PPV3-DNA was detected in H4 in 11.12% of litters (4/36) at weeks 1 and 3 of age, with two litters positive at week 1 and two at week 3. Consistent with the earlier phases of the study (PP and FP), we did not detect PCV4 or PPV8 throughout the LP. Viral loads and Ab titers for each virus in litters from PS and MS are detailed in Table 3 and Figure S5. Further results for each herd are summarized in Table S11 and Figure S7.

No significant differences were found between the PS and MS regarding PCV2 viral loads and PCV2-Ab titers (*p* = 0.8). PCV2-DNA was detected in 88.89% (32/36) of pre-suckling serum samples. However, this proportion decreased to 36.12% (13/36) by week 3 of age (Table 3). The mean PCV2 viral load in the LP was 4 log 10 copies (Ct > 30.3), indicating a subclinical infection. In terms of anti-PCV2 Abs, they were present in 61.12% (22/36) of pre-suckling sera and increased significantly (*p* < 0.01) at weeks 1 and 3, reaching 100% (36/36). For PCV3, we also did not observe significant differences (*p* = 0.7) between the litters from PSs and MSs concerning viral loads or anti-PCV3 Abs. In the pre-suckling serum samples, PCV3-DNA was detected in 11.12% (4/36) of the litters (H2 and H3). This percentage increased to 41.67% (15/36) at week 3 of age (Table 3). The viral loads of PCV3 did not exceed 3.9 log 10 copies/mL (Ct > 30.7), which suggests a subclinical infection. Anti-PCV3 Abs were detected in the pre-suckling serum in 8.34% (3/36) of litters (H2 and H3). Serological testing for anti-PCV3 Abs was not performed in piglets at weeks 1 and 3 of age. In the context of PPV1, no significant differences (*p* = 0.6) were found in viral loads between PS and MS litters. PPV1-DNA was detected in 27.78% (10/36) of pre-suckling serum samples; by week 3 of age, 25% (9/36) of litters remained viremic. For Abs, there was a significant increase (*p* < 0.01), with pre-suckling levels at 13.89% (5/36) in MS litters rising to 55.56% (20/36) by week 3 of age. The anti-PPV1 Ab titers were significantly higher (*p* < 0.01) at week 1 compared to pre-suckling titers, and these elevated titers persisted until week 3. Regarding PRRSV, we found no significant differences in viral loads or Abs titers between PS and MS litters (*p* = 0.6). During the LP, PRRSV-RNA was detected only in H1, where 11.12% (4/36) of the litters were viremic at delivery (pre-suckling sera) and continued to be viremic until week 3 of age. Additionally, there were no significant differences (*p* = 0.9) in viral loads across the three LP time points evaluated (Table 3) in H1 PRRSV-positive litters. Regarding anti-PRRSV Abs, all litters were seronegative at pre-suckling, but 33.34% (12/36) seroconverted by weeks 1 and 3 of age, specifically (10/36) in H1 and (2/36) in H3.

#### Viral Coinfections During the Lactation Phase

**Figure 7:**
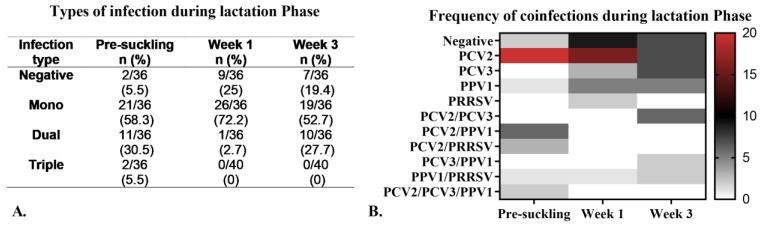
Types of infections observed during the lactation phase (LP). (**A**). Frequency of infection types (mono- and coinfections). (**B**). Heat map displaying the frequency of coinfections during the LP. The lateral scale ranges from 0 to 20, indicating the number of infections, where red represents the highest frequency, black denotes the average frequency, and white indicates the absence of the virus.

Figure 7A illustrates the percentage of positivity for each type of infection throughout the LP. In the pre-suckling serum, we found that 5.55% (2/36) of samples tested negative for all viruses. Among the positive samples, mono-infections were the most frequent (58.3%), followed by dual coinfections (30.5%). PCV2 was the most commonly detected virus in mono-infection, with a decreasing frequency of 95% at pre-suckling, 61% at week 1, and 36% at week 3 of age (Figure 7B). Various dual coinfections were also identified, with PCV2/PPV1 being the most common in pre-suckling serum and at week 1 of age, while PCV2/PCV3 was the most frequent at week 3 (Figure 7B). We did not find a significant association (*p* = 0.75) between coinfections and the viral load for any primary virus (Table S12).

### 3.5. Histopathological Examination of Tissues Collected During Delivery

Histopathological examination of placental tissues (*n* = 37) showed that 86.49% (32/37) exhibited mild to severe multifocal congestion, 75.68% (28/37) displayed multifocal hemorrhagic foci, and 62.17% (23/37) had multifocal degenerative changes in the chorionic epithelium (Figure S8A). These lesions were more commonly found in cases of PCV2 mono-infection and PCV2/PPV1 coinfection; however, no significant differences (*p* = 0.7) were observed between the two types of infection (Figure S8A). For umbilical cords (*n* = 37), the primary lesion identified was multifocal hemorrhages, which were present in 100% of samples. Additionally, degenerative changes in the amniotic epithelium were noted in 13.52% (5/37), particularly in PCV2/PPV1 coinfection (Figure S8B). Regarding fetal tissues, histopathological analysis revealed that 43.38% (36/83) of hearts were lesion-free. The most common myocardial lesion was mononuclear cell infiltration, occurring in 24.10% (20/83) of cases, and was observed across all infection types, with a higher frequency in PCV2/PRRSV coinfection (Figure S8C). In lungs (*n* = 82), 48.78% (40/82) showed congestion of the interalveolar septa, predominantly associated with PCV2/PPV1 coinfection. The second most common finding was mild thickening of the alveolar septa, noted in 15.86% (13/82) of lung samples, which was more frequent in PCV2/PRRSV coinfection. No lesions were found in 20.74% (17/82) of fetal lungs (Figure S8D). Regarding the histopathology of other fetal tissues, including the liver, brain, spleen, and kidney, analysis was conducted only on stillborn cases due to the significant autolysis seen in mummies. The liver (*n* = 46) exhibited extramedullary hematopoiesis as the most common lesion, while various spleen regions (*n* = 46) also showed hematopoiesis. Brain tissue (*n* = 46) frequently presented signs of congestion and gliosis, and renal congestion was observed in 80.86% (38/47) of kidney samples. The lesions found in stillborns were consistent across all types of infection, indicating that they were not specific for any mono- or coinfection. Table S13 provides detailed information on the frequency of lesions in each evaluated tissue and their association with the type of infection.

### 3.6. Genetic Analysis of the Viruses

The number of sequences obtained in this study was limited due to low viral loads in the samples. For PCV2, four ORF2 sequences (701 nt) were retrieved, with Ct values ranging from 29 to 30. One sequence was sourced from a mummy from H3, while three sequences originated from H2, associated with a stillborn and two placentas. Phylogenetic analysis indicated a high similarity of 99.3 to 99.8% with PCV2 sequences reported in Colombia in 2021, placing them within the PCV2d genotype (Figure S9A). For PCV3, one ORF2 sequence (645 nt) was retrieved from a mummified fetus (Ct = 18), showing 99.2 to 99.8% identity with previously reported PCV3 sequences from Colombia, categorizing it within the PCV3a genotype (Figure S9B). In the case of PRRSV, one ORF5 sequence (602 nt) was obtained from a stillborn (Ct = 29.5), demonstrating 99% identity with strains previously reported in the same province and herd in Colombia. This sequence was categorized within lineage 1, sublineage A, exhibiting a restriction enzyme cleavage pattern (RFLP) of 1-7-4 (Figure S9C). For PPV1, no sequences were retrieved.

### 3.7. Proposed Diagnostic Criteria for Subclinical Porcine Reproductive Failure

After years of research on the primary PRF viruses, a consensus has been reached regarding the clinical case definitions for each one. For PCV2, the clinical signs linked to PRF include irregular return to estrus, abortions, and mummied fetuses. The individual diagnostic criteria for PCV2 include PRF in late gestation, fibrin–necrotic myocarditis in fetuses, and moderate to high viral loads in the myocardium [35]. For PCV3, the clinical signs associated with PRF involve late-term abortions, mummified fetuses, malformations, stillbirths, and weak piglets at birth. Individual diagnostic criteria include lymphoplasmacytic to multisystem lymphohistiocytic perivascular inflammation and moderate to high viral loads in the fetus [36]. Finally, for PRRSV, the clinical signs associated with PRF are infertility, abortions, premature births, increased stillbirths, and weak piglets at birth [37]. A high viral load in meconium-stained stillbirths has been established as an individual diagnostic criterion [38]. A common consensus for establishing a clinical case of PRF related to these viruses is detecting high viral loads in fetal tissues through quantitative PCR (qPCR). Our study examined PS, MS, and their offspring during three reproductive phases: pregnancy, farrowing, and lactation. The findings revealed the presence of several viruses associated with PRF, specifically PCV2, PCV3, PPV1, and PRRSV, at low viral loads in both sows and fetuses. Despite these low loads, there were moderate to high alterations in reproductive parameters. This suggests that subclinical infections may be present in both sows and fetuses, potentially affecting PRF in ways not identified by conventional diagnostic criteria. In light of this observation, we propose the criteria outlined in Table 4 to identify and differentiate subclinical infection related to PRF for these four viruses.

## 4. Discussion

In this longitudinal study, we monitored 40 sows (18 PSs and 22 MSs) and their offspring throughout the pregnancy, farrowing, and lactation phases. Our results showed the presence of four viruses, PCV2, PPV1, PCV3, and PRRSV, with PCV2 being the most frequently detected during all three phases. We made two significant findings regarding the viral infections detected in the study: (i) We identified the four viruses studied that exhibited low viral loads, suggesting the presence of subclinical infections with these pathogens. However, the reproductive parameters indicate potential SMEDI, particularly for H1, H2, and H3. This finding may be caused by a synergistic effect of the primary PRF viruses on subclinical viral loads or by factors not evaluated, such as other pathogens or herd management practices. (ii) We observed both mono- and coinfections and subclinically throughout the three phases. Our findings prompted us to propose criteria for categorizing subclinical reproductive infections caused by PCV2, PPV1, PCV3, and PRRSV, including their coinfections. However, this initial proposal needs further validation through additional research.

In this study, PCV2 was detected at high rates across all three phases (PP, FP, and LP), even though routine vaccination was implemented in all four evaluated herds. This observation contrasts with other studies that indicated a low frequency of PCV2 in vaccinated herds [40,41,42]. There is a consensus that PCV2 immunization reduces infection rates [43] and lowers the prevalence of PRF [40] when following recommended vaccination regimens. Moreover, vaccinating sows at different time points, similar to blanket vaccination, improves productive parameters and maintains low levels of viremia [44]. Our study revealed the continuous and low-level (subclinical) viral circulation of PCV2 despite the routine vaccination of both PS and MS. We found no significant differences in the frequency of PCV2 presentation between these two groups, which aligns with recent research [15,45,46]. Additionally, prior studies have suggested that vaccinating sows against PCV2 might positively influence the prolificacy and vitality of the offspring in subclinically infected herds while also revealing non-PCV2-related lesions in fetuses [47]. We supported the view that PCV2 vaccination programs have altered the viral epidemiology in two ways: first, by creating immunologically susceptible subpopulations within herds [39], and second, by lowering viremia levels, which allows the remaining viral population to lead to subclinical infections [48]. Our study revealed that vaccination of the sows did not prevent vertical transmission [49,50], as indicated by detecting PCV2-DNA in pre-suckling serum, fetal tissues, and colostrum. Nevertheless, PCV2 viral loads were maintained at low levels (<4 log10 copies/mL or g) throughout all phases, with minimal or absent histopathological lesions. These findings suggest that a subclinical reproductive form of PCV2 infection is the predominant pattern in vaccinated populations. Additionally, the presence of anti-PCV2 Abs at insemination and farrowing, alongside continued viral circulation, indicates a non-sterilizing immune response induced by current PCV2 vaccines [51].

For PCV3, we observed low positivity rates along with low viral loads (<4 log10 copies/mL) during all three phases of our study, which contrasts with previous studies that reported high viral loads of PCV3 [52,53]. Our findings identified the highest PCV3 positivity at the time of insemination, which contradicts earlier studies that found it more frequently at delivery [52]. Similar to other research, we detected PCV3-DNA in some pregnant sows for over 40 days, reinforcing the hypothesis that PCV3 can establish persistent infections [54]. Unlike prior studies that reported a higher frequency of PCV3 in gilts [52,55], our research did not show a significant increase in PCV3 positivity or viral load between PS and MS. We found higher anti-PCV3 Ab titers in MSs compared to PSs, supporting earlier reports that indicated sows have more anti-PCV3 Abs than gilts [56]. At the fetal level, the highest PCV3 viral loads were detected in fetuses with a CRL < 17 cm, suggesting that primary infection occurs during the first half of pregnancy. Two hypotheses may explain the lower viral loads observed in older fetuses (CRL > 17 cm): (i) higher susceptibility to PCV3 in the early stages of development, as recently described by [57], and (ii) a decreased viral load in older fetuses due to the development of immunocompetence after day 70 of pregnancy, similarly to mechanisms reported for PCV2 [58]. The establishment of low viral loads throughout the three phases of the investigation (pregnancy, farrowing, and lactation) and the absence of distinctive histopathological lesions support the idea of a subclinical reproductive infection pattern of PCV3, akin to that of PCV2 [54,59]. Regarding the potential impact of PCV3 infection on the efficacy of PCV2 vaccination, our study’s consistently low PCV2 viral loads observed throughout all three phases indicate that such an effect likely did not occur. However, we cannot entirely dismiss the possibility of a synergistic effect between these two viruses on PRF. Recent research has also explored how PCV3 circulation affects the efficacy of the PCV2 vaccine. These studies have found no impact; even in animals with high PCV2 loads, the level of PCV3 infection was not increased [60]. This suggests that the potential synergistic effect between these two viruses remains unclear.

Phylogenetic analysis of PCVs revealed that the genotypes PCV2d and PCV3a were identified in the four herds studied. This finding aligns with recent research conducted over the past five years in Colombia, where these two genotypes have been the most commonly detected [53,61,62,63,64]. Additionally, consistent with our previous research [62,63], PCV4 was not detected in this study. However, it is important to contextualize this finding with existing literature. Previous studies have reported varying prevalence rates of PCV4 in pigs of different ages [65,66] and in various types of tissues [29]. Specifically, in the reproductive context, PCV4 has been found in serum from pregnant sows as well as in aborted fetuses [65,66]. These findings highlight the necessity for continued surveillance and further investigation into the potential role of PCV4 in PRF.

PPV1 was identified in all three phases of the study despite routine vaccination. This finding contrasts with previous studies that demonstrated vaccination as effective in controlling economic losses and reducing prevalence [67,68]. We observed PPV1-viremic sows throughout their pregnancy and during farrowing. Additionally, we detected PPV1-DNA in fetuses and both the viral genome and Abs in pre-suckling sera, suggesting that vaccination does not completely inhibit vertical transmission, as previously reported [69]. Significant differences in Ab titers were noted between the PS and MS, with PSs and piglets exhibiting lower Abs levels. These findings are consistent with earlier studies that reported lower Ab titers in gilts compared to sows [70], which may be attributed to individual immunological factors [71] and the antigenic properties of the vaccines [71,72]. Despite the general agreement on the efficacy of PPV1 vaccination, the emergence of highly virulent PPV1 strains, characterized by limited cross-reactivity to vaccine strains [73], suggests that current vaccines may not provide optimal protection against new variants [73]. This is particularly relevant in Colombia, where the circulation of the 27a strain has been documented [74]. However, we could not confirm its presence in this study because low viral loads prevent sequencing. Our findings suggest that current vaccination confers partial protection by reducing viral loads but does not prevent infection. The absence of distinctive lesions in the fetuses confirms subclinical infection, highlighting the need to characterize circulating PPV1 strains and, if necessary, optimize vaccination strategies. 

This study revealed contrasting results regarding PRRSV among the four herds evaluated. In one herd, PRRSV-RNA and Abs were detected, indicating an unstable infection status according to established criteria [75]. Our findings showed that PRRSV infection occurred during early pregnancy, as evidenced by the detection of PRRSV-RNA (PS and MS sera) and Abs in 100% of the sows in the PRRSV-positive herd during PP. While the literature suggests that effects of PRRSV are primarily observed in the last third of pregnancy [76,77], our study identified PRRSV-RNA in mummies with various CRLs (5 to 28 cm). This aligns with reports indicating that PRRSV infection can occur at any stage of pregnancy [78]. In the PRRSV-positive herd, all viremic sows exhibited low viral loads (<3.5 log copies/mL) and demonstrated varied effects on vertical transmission. Some sows did not transmit the virus to their progeny, while others did, resulting in differing PRRSV viral loads in the litters. Live piglets were also found to be viremic, with low viral loads (<3 log copies/mL), whereas stillborn piglets exhibited the highest viral loads (up to 6 log copies/g), aligning with previous studies [38]. We identified the circulation of PRRSV-2 lineage 1A, which exhibited RFLP 1-7-4. This viral strain emerged in the USA in 2014 and has significantly impacted North American swine production [79]. This is the first report on these strains in Colombia, and it raises questions regarding transmission routes and the biosecurity measures implemented on farms. In contrast, the second PRRSV-positive herd demonstrated a stable positive infection pattern [75], characterized by the presence of Abs without detection of the viral genome. The absence of clinical signs and viremia in all sows and their progeny indicates effective passive immunity without evidence of vertical infection. This demonstrates that PRRSV infection can be controlled through adequate passive immunity [80] and strict biosecurity measures. These practices are particularly important in Colombia, where PRRSV vaccination is not available.

We identified several nPPVs, specifically PPV2, PPV3, PPV4, and PPV6, in serum samples from sows (PS and MS) throughout the PP. These viruses were subsequently found in the sera of their litters during the LP, albeit at low viral loads. Importantly, these nPPVs were not detected in fetal or maternal tissues or colostrum. Our results do not confirm or rule out the potential effects of these viruses on the PRF; they may circulate within breeding sow populations without causing reproductive pathology. These findings align with previous studies that reported the detection of these viruses in the serum of breeding sows and fetal tissues [81], as well as studies noting a high prevalence of PPV3, PPV5, and PPV6 in serum samples from replacement gilts. This suggests a possible impact when these viruses are coinfected with PCV2, PCV3, and PRRSV [63].

In our investigation into coinfections, we identified dual and triple viral concurrences across the three evaluated phases. In PP and LP, the most common coinfections among sows were PCV2/PPV1 and PCV2/PCV3; meanwhile, in fetuses (during FP), the predominant coinfections were PCV2/PPV1 and PCV2/PRRSV. This study demonstrates that PCV2/PPV1 coinfection influenced PCV2 replication, as evidenced by significantly higher PCV2 viral loads in both PS and MS serum samples at delivery and their fetuses, compared to PCV2-positive mono-infection. This observation suggests a synergistic effect between PPV1 and PCV2, consistent with previous reports in pigs suffering from PMWS, where PPV1 exacerbated disease severity in PCV2-infected pigs [82]. Furthermore, the coinfection PCV2/PPV1 in fetuses has been extensively documented [12,83,84,85] concerning mummified fetuses with various CRLs [12], more severely histopathological lesions [14,86], and weak-born piglets [15]. The coinfection PCV2/PRRSV was found in 16.8% of fetuses, which supports earlier studies [12,13,45,87]. The current study revealed an interesting finding: the probability of detecting PRRSV decreased in fetuses that were also positive for PCV2, PCV3, or PPV1. This phenomenon is not well understood. However, some estimates from a previous study indicated that fetal coinfections involving PRRSV did not significantly affect reproductive outcomes [38]. While we did not observe any differences in viral loads for any of the viruses in fetuses or sows with PCV2/PRRSV coinfection, we did find significant lower anti-PCV2 Ab titers in the coinfected sows. This might be due to the shared affinity of both viruses for immune system cells [88,89].

Throughout the LP, we observed decreased PCV2-DNA detection across all evaluated litters, accompanied by an increase in maternal anti-PCV2 Abs up to week 3. This finding reinforces the importance of passive immunity in controlling viral infection [90]. In contrast, 50% of the litters did not detect maternal PPV1-Abs. In the litters that did show PPV1 Abs, the levels decreased on average by week 3. Previous research has established that the levels of maternal PPV1-Abs in piglets are correlated with the serum Abs levels of the sow [72], which aligns with our findings since not all sows seroconverted. During the LP period, coinfections occurred at low frequency, with PCV2/PPV1 predominating in the pre-suckling phase and week 1, before switching to PCV2/PCV3 by week 3. This indicates varying patterns of viral infection during the early weeks of the piglet’s life. There were no significant changes in viral loads or Ab in litters experiencing coinfections, suggesting that maternally derived Abs may have a protective effect, consistent with previous reports on viral mono-infections [91,92,93].

Given that reproductive parameters were affected in herds H1, H2, and H3, it is necessary to consider that, although the viral loads of the studied viruses were low and classified as subclinical infections, there may be a synergistic effect among the primary PRF viruses at these low levels, potentially resulting in SMEDI. The established understanding of viral coinfections suggests that one virus can either enhance the robustness of another virus or inhibit its replication [17]. However, the occurrence of a synergistic effect from two or more viruses, even with subclinical viral loads that lead to clinical signs, somewhat contradicts this traditional view. This idea is not entirely new, as it has been previously proposed in studies of virus/bacterial coinfections in human respiratory diseases [94]. In this study, we observed altered reproductive parameters in at least three of the four farms evaluated, indicating the presence of SMEDI. Our results, which showed the subclinical concurrence of several viruses, suggest potential pathways that may lead to a synergistic effect, ultimately resulting in the manifestation of PRF. The primary virus suspected to be crucial to this effect is likely PRRSV. This assumption is supported by detecting PCV2, PPV1, and PCV3 subclinically in all four herds studied. However, PRRSV was only found in one herd (H1) as an active infection and in another (H3) as a previous infection, as indicated by the presence of Abs. In the herd with active PRRSV infection, we recorded significant impacts on reproductive parameters, with a percentage of mummies of 20% and a stillbirth rate of 9.4%. We also identified coinfections involving PRRSV, such as PCV2/PRRSV and PCV3/PRRSV, during PP in serum samples. In contrast, for dual infections during FP, we identified PCV2/PRRSV (small and large mummies), PCV3/PRRSV (small mummies), and PPV1/PRRSV (small mummies), along with a triple infection PCV2/PPV1/PRRSV (small mummies). These findings suggest PRRSV synergizes with other PRF viruses, inducing PRF even at low viral loads. Previous research has linked reproductive parameters to low PRRSV viral loads [63], but this is the first study to propose such a synergistic effect. This potential synergistic effect observed in the herd infected with PRRSV was an important aspect of the study. Notably, the highest positivity rates for PCV3 and PCV2 were detected at delivery, clarifying that these remained at subclinical loads. Furthermore, PRRSV appears to be the main contributor to this synergistic effect. This is supported by findings from herd H3, where PRRSV-Abs were detected without the virus (H3), indicating that the herd had stabilized after a previous infection. This herd exhibited better reproductive parameters, such as a lower percentage of mummies and stillborns, than an unstable herd. The second option involved cases where PRRSV was absent. The possibility of a synergistic effect involving the other primary PRF viruses remains uncertain. The results varied among three herds without PRRSV: two herds experienced SMEDI, while one did not. The differences between these herds were challenging to pinpoint, as the detected viruses exhibited similar viral loads and Abs levels. The findings suggest that there may be a greater variety of subclinical coinfections in herds that experienced SMEDI.

## 5. Conclusions

This longitudinal study offers valuable new findings into the complex dynamics of porcine viral infections during pregnancy, farrowing, and lactation phases. Despite the implementation of vaccination strategies, we observed a significant frequency of subclinical infections. We also identified different patterns of vertical transmission and characterized viral coinfections, exploring their potential synergistic effects. Our findings emphasize the need for updated diagnostic criteria for subclinical reproductive infections.

## Figures and Tables

**Table 1:** Comparative analysis of PCV2, PCV3, PPV1, and PRRSV genome copies (mean log 10 copies/mL) as well as antibody response (mean IgG ELISA values) in primiparous and multiparous sows during the pregnancy phase.

	Sows	Insemination		Day 40	Day 80	Delivery	
Virus		Antibodies S/P (±), *n*	Viral Loads Log Copies (±), *n*	Viral Loads Log Copies (±), *n*	Viral Loads Log Copies (±), *n*	Antibodies S/P (±), *n*	Viral Loads Log Copies (±), *n*
PCV2	Primiparous	0.9 (0.21) 18/18	3.9 (0.6) 6/18	3 (0.0) 1/18	3.8 (0.4) 8/18	1.2 (0.32) 18/18	3.8 (0.76) 15/18
	Multiparous	0.91 (0.25) 22/22	4 (0.67) 8/22	4.25 (0.0) 1/22	4.2 (0.71) 9/22	1.29 (0.41) 20/20	3.8 (0.82) 18/20
PCV3	Primiparous	NP	3.8 (1) 7/18	4.2 (0.25) 7/18	3.6 (0.3) 8/18	0.23 (1.4) 6/18	4 (0.4) 2/18
	Multiparous	NP	3.8 (0.74) 8/22	4 (0.21) 8/22	3.65 (0.51) 10/22	1.3 (0.98) * 13/20	4 (0.15) 3/20
PPV1	Primiparous	0.76 (0.41) 12/18	3.7 (0.14) 10/18	4 (0.4) 3/18	3.7 (0.05) 3/18	0.34 (0.3) 8/18	3.7 (0.56) 7/18
	Multiparous	0.95 (0.4) 14/22	3.8 (0.16) 9/22	3.8 (0.17) 4/22	4 (0) 1/22	0.7 (0.36) * 13/20	3.76 (0.23) 6/20
PRRSV	Primiparous	0.01 (0.56) 5/18	0/18	3 (0.2) 4/18	3 (0.2) 4/18	0.21 (0.64) 7/18	3 (0.0) 1/18
	Multiparous	0.02 (0.7) 5/22	0/22	3 (0.2) 4/22	3 (0.0) 3/22	0.07 (0.39) 6/20	3 (0.11) 3/20

NP: not performed; (±) represents the standard deviation; * statistically significant differences between primiparous and multiparous sows (*p* < 0.05); *n*: positives/total samples.

**Table 2:** Viral loads and viral frequencies of PCV2, PCV3, PPV1, and PRRSV in samples from primiparous and multiparous sows during the farrowing phase (FP).

Virus	Sows	Placenta Viral Load Log 10 Copies (±), *n*	Umbilical Cord Viral Load Log 10 Copies (±), *n*	Colostrum Viral Load Log10 Copies (±), *n*
PCV2	Primiparous	4 (0.23), 12/18	4 (0.3), 14/18	3.9 (0.5), 12/18
	Multiparous	4.2 (0.57), 15/19	4.2 (0.6), 18/19	4.1 (0.43), 15/19
PCV3	Primiparous	4 (0.26), 4/18	3.7 (0.23), 4/18	4 (0.6), 6/18
	Multiparous	3.7 (0.01), 4/19	3.7 (0.1), 5/19	3.7 (0.16), 5/19
PPV1	Primiparous	3.9 (0.37), 8/18	4 (0.59), 9/18	3.8 (0.64), 6/18
	Multiparous	3.8 (0.32), 8/19	3.9 (0.39), 6/19	3.95 (0.44), 7/19
PRRSV	Primiparous	3 (0.0), 1/18	3 (0.0), 1/18	3 (0.0), 1/18
	Multiparous	3 (0.75), 4/19	3 (0.46), 3/19	3.4 (0.56), 2/19

±: Standard deviation, *n*: positives/total samples.

**Table 3:** Viral loads and antibody titers for PCV2, PCV3, PPV1, and PRRSV in serum samples from litters of primiparous and multiparous sows throughout the lactation phase.

		Pre-Suckling		Week 1		Week 3	
Virus	Sows	Antibodies S/P (±), *n*	Viral Load Log Copies (±), *n*	Antibodies S/P (±), *n*	Viral Load Log Copies (±), *n*	Antibodies S/P (±), *n*	Viral Load Log Copies (±), *n*
PCV2	Primiparous	0.39 (0.28), 12/17	4 (0.48), 14/17	1.2 (0.35), 17/17	4 (0.32), 8/17	1.16 (0.33), 17/17	3.7 (0.34), 6/17
	Multiparous	0.3 (0.35), 10/19	4 (0.4), 18/19	1.3 (0.38), 19/19	3.7 (0.52), 8/19	1.14 (0.37), 19/19	3.4 (0.35), 7/19
PCV3	Primiparous	0.05(0.28), 2/17	3.4 (0.0), 1/17	NP	3.5 (0.21), 2/17	NP	3.7 (0.8), 10/17
	Multiparous	0.04 (0.27), 1/19	3.8 (0.57), 3/19	NP	3.55 (0.21), 2/19	NP	3.4 (0.34), 5/19
PPV1	Primiparous	0.09 (0.09), 4/17	3.8 (0.2), 5/17	0.58 (0.38) *, 11/17	4.4 (0.23), 4/17	0.25 (0.38) *, 8/17	4 (0.1), 4/17
	Multiparous	0.06 (0.06), 1/19	3.8 (0.13), 5/19	1.01 (0.4) 15/19	4.3 (0.0), 1/19	0.93 (0.43), 12/19	4 (0.5), 5/19
PRRSV	Primiparous	0.02 (0.01), 0/17	5 (0.0), 1/17	0.05 (0.45), 6/17	3.7 (0.0), 1/17	0.03 (0.3), 6/17	3 (0.0), 1/17
	Multiparous	0.03 (0.0), 0/19	3.7 (0.25), 3/19	0.03 (0.56), 6/19	4.05 (0.21), 2/19	0.1 (0.41), 6/19	3 (0.0), 1/19

NP: Not performed, (±): standard deviation, *: statistically significant differences between primiparous and multiparous sows (*p* < 0.05); *n*: number of litters positive/total litters.

**Table 4:** Proposed criteria to distinguish between reproductive disease (symptomatic) and subclinical reproductive infection (asymptomatic) in sows infected with viruses involved in porcine reproductive failure (PCV2, PCV3, PPV1, and PRRSV). The criteria for subclinical infections are based on the findings from this study.

Virus	Reproductive Disease	Reproductive Subclinical Infection
PCV2	Reproductive failure or SMEDI, return to estrus Fibrous to necrotizing myocarditis of fetuses Moderate (>5 log10 copies) to high (>7 log10 copies) viral load of PCV2 in tissues samples of mummies, stillborns and newborn piglets Seroconversion following return to estrus PCR or qPCR positivity detected during irregular return to estrus [39]	Lack of overt reproductive clinical signs or SMEDI No or minimal histopathological lesions in fetal tissues Low PCV2 viral loads (<4 log10 copies) by qPCR or ISH in fetal and maternal tissues (fetal hearth and placenta) Low PCV2 viral loads (<4 log10 copies) by qPCR in sows during pregnancy and farrowing Detection of pre-suckling PCV2-Abs and low PCV2 viral load (<4 log10 copies)
PCV3	Late reproductive signs (abortion, malformations, mummified fetuses, stillborn fetuses, weak-born piglets) and higher perinatal mortality Multisystemic lymphoplasmacytic to lymphohistiocytic perivascular inflammation Focal myocardial necrosis with mononuclear infiltration in stillborn and mummies Moderate to high (>10^5^) PCV3 viral loads in damaged tissues [36]	Lack of overt reproductive clinical signs or SMEDI No or minimal lesions in fetal tissues Low PCV3 viral loads (<4 log10 copies) in fetal and maternal tissues Seropositivity in sows during farrowing and low PCV3 viral loads (<4 log10 copies) in sows during pregnancy by qPCR Detection of pre-suckling PCV3-Abs and low PCV3 viral loads (<4 log10 copies)
PPV1	Reproductive failure or SMEDI PPV1 Ag in tissues [4] High (>5 log10 copies) viral load of PPV1 in fetuses	Lack of overt reproductive clinical signs or SMEDI No or minimal lesions in fetal tissues Low PPV1 viral loads (<4 log10 copies) in fetal and maternal tissues Detection of pre-suckling PPV1-Abs and low PPV1 viral loads (<4 log10 copies) Low PPV1 viral loads (<4 log10 copies) in sows during pregnancy and farrowing by qPCR
PRRSV	Late Reproductive failure or SMEDI High PRRSV viral loads (>4 log10 copies) in reproductive lymph nodes of sows Moderate to high PRRSV viral loads (>4 log10 copies) in meconium-stained fetuses [38]	Lack of overt reproductive clinical signs or SMEDI Low PRRSV viral loads (<3.5 log10 copies) in sows during pregnancy and farrowing by qPCR PRRSV resilient litters: low percentage of fetuses dead and low (<3.5 log10 copies) or negative PRRSV-RNA levels [38]

## Data Availability

All required data are available as texts and figures in the main text and the article’s Supplementary Materials.

## Supplementary Materials

The following supporting information can be downloaded at https://www.mdpi.com/article/10.3390/pathogens14060573/s1: Figure S1. A. A Comparison of reproductive parameters between gilts (Gs) and sows (Ss) in the study population. B. Reproductive performance of gilts and sows across four distinct herds. Figure S2. Viral circulation during the pregnancy phase (PP) in gilts and sows. Figure S3. Viral circulation during pregnancy phase across individual herds, illustrating Ab levels (bars) and viremia (symbols). Figure S4. Viral detection rates and viral loads (Log10 copies/g) in mummified fetuses and stillborn piglets from individual herds. Figure S5. Boxplot depicting viral loads in fetuses for PCV2, PCV3, PPV1, and PRRSV based on the type of infection: mono-infection (green), dual infection (red), and triple infection (blue). Figure S6. Viral circulation during the lactation phase (LP) in litters from gilts and sows. Figure S7. Viral circulation during the lactation phase (LP) across individual herds, showing Ab levels (bars) and viremia (symbols). Figure S8. Heat map that illustrates the frequency of histopathological lesions found in tissues collected at delivery from gilts, sows, and fetuses, along with their association with different types of infection. Figure S9. Maximum likelihood (ML) phylogenetic trees for PCV2-ORF2, PCV3-ORF2, and PRRSV-ORF5 were estimated based on the alignment of the nucleotide sequences. Table S1. Characteristics of the evaluated pig herds in this study, including the reproductive parameters of the monitored gilts (primiparous sows) and sows (multiparous sows) over time, as well as details of the collected samples. Table S2. List of primers used in the present study for virus detection. Table S3. Standardized Real-time PCRs details for identifying and quantifying PCV2, PCV3, PPV1, and PRRSV in this study. Table S4. ELISA results and qPCR viral loads for PCV2, PCV3, PPV1, and PRRSV at insemination and delivery across the four evaluated herds (H1 to H4). Table S5. Mean viral loads (log 10 copies/mL) for PCV2, PCV3, PPV1, and PRRSV in different types of infection (mono- and coinfections) detected during the pregnancy phase (PP). Table S6. Viral load and positivity for PCV2, PCV3, PPV1, and PRRSV in the placenta, umbilical cords, and colostrum across the four herds evaluated (H1 to H4) during the farrowing phase (FP). Table S7. Number and percentage of infection types (mono and coinfections) detected in fetal samples (mummies and stillborns). Table S8. Frequency of PRRSV, PCV2, PCV3, and PPV1 in mummified fetuses and stillborns across different herds. Table S9. Mean Viral loads (log 10 copies/mL) established for different types of infections in fetal samples. Table S10. Multivariate analysis of associations between detected viral infections (PCV2, PCV3, PPV1, and PRRSV) in fetuses (dependent variables) and coinfection status, parity, and vaccination against PCV2 and PPV1 (independent variables). Table S11. Results from ELISA and viral loads for PCV2, PCV3, PPV1, and PRRSV during the lactating phase (LP) in each evaluated herd. Table S12. Average viral loads (log 10 copies/mL) for different types of infections during the lactation phase (LP). Table S13. Frequency of histopathological findings in maternal and fetal tissues established according to different types of infection (mono- and coinfections).

## Abbreviations

The following abbreviations are used in this manuscript:

PRF	Porcine reproductive failure
SMEDI	Stillbirth, mummification, embryonic death, and infertility
PCV2	Porcine circovirus type 2
PPV1	Porcine parvovirus 1
PRRSV	Porcine respiratory and reproductive syndrome
PCV3	Porcine circovirus type 3
PPV	Porcine parvovirus
H	Herd
PSs	Primiparous sows
MSs	Multiparous sows
PP	Pregnancy phase
FP	Farrowing phase
LP	Lactation phase
Abs	Antibodies
PBA	Piglet born alive
CRL	Crown-rump length
PWM	Preweaning mortality
nPPVs	Novel porcine parvovirus
