# Supplementary material for: Longitudinal Monitoring of Mono- and Coinfections Involving Primary Porcine Reproductive Viruses (PCV2, PPV1, and PRRSV) as Well as Emerging Viruses (PCV3, PCV4, and nPPVs) in Primiparous and Multiparous Sows and Their Litters
Source: Pathogens. 2025 Jun 7;14(6):573. doi: 10.3390/pathogens14060573 (PMC12196009; doi:10.3390/pathogens14060573)
Supplement: Supplementary file 1 [file pathogens-14-00573-s001.zip › pathogens-3631506-supplementary.pdf]

Supplementary Materials

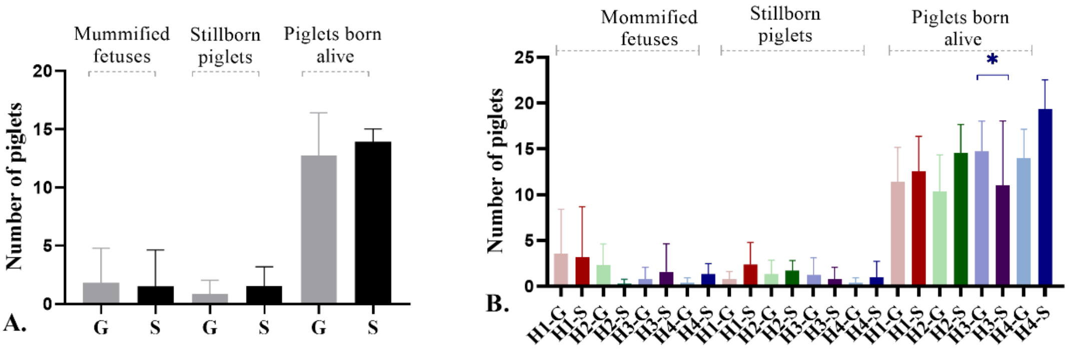

Figure S1. A. A Comparison of reproductive parameters between gilts (G) and sows (S) in the study population. B. Reproductive performance of gilts and sows across four distinct herds. The parameters evaluated include MM (mummified fetuses), NS (number of stillborn piglets), and PBA (number of piglets born alive). The data is color-coded as follows: Herd 1 (H1) is represented in red, Herd 2 (H2) in green, Herd 3 (H3) in Purple, Herd 4 (H4)in Blue. Asterisks (*) indicate significant differences (p <0.05) between gilts and sows within each herd. **Key finding:** At the herd level, a significantly higher number of PBA was observed in sows from Herd 4 (p <0.05).

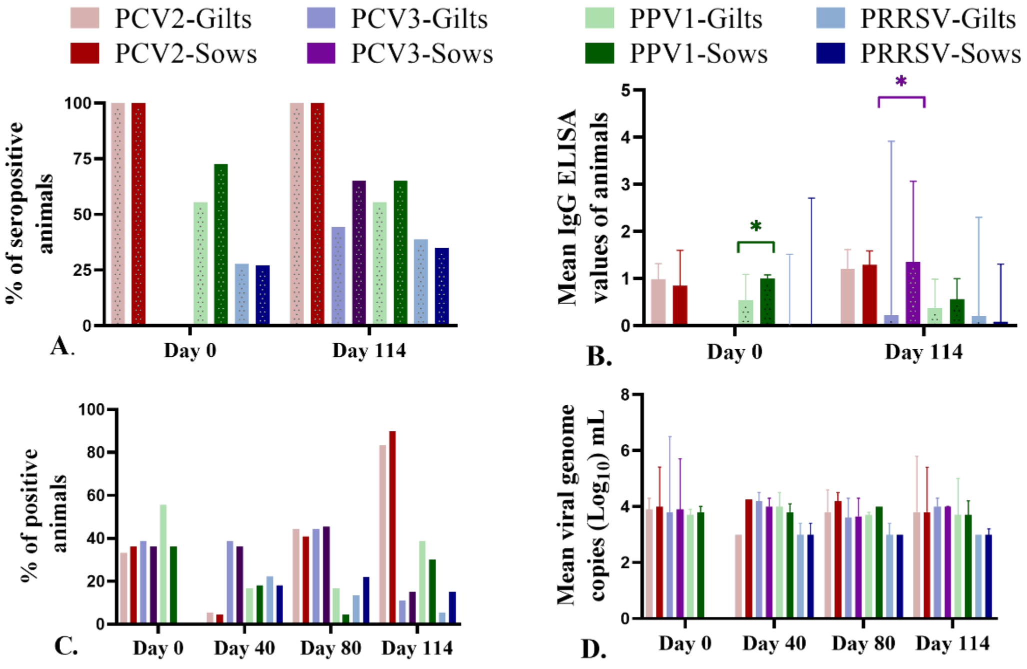

Figure S2. Viral circulation during the pregnancy phase (PP) in gilts and sows. This analysis examines four primary reproductive viruses, each represented by distinct colors: PCV2 in red, PPV1 in green, PRRSV in blue, and PCV3 in purple. The figure consists of four panels, each illustrating a different aspect of viral circulation: A. Percentage of seropositivity in gilts and sows, B. Mean IgG ELISA values for gilts and sows, C. Percentage of viral-positive sows or gilts, D. Mean viral genome copies (log10 / mL) * p <0.05 indicates significant differences between gilts and sows. **Key finding:** There are differences in antibody (Ab) levels between primiparous and multiparous sows for PPV1 and PCV3 (p <0.05).

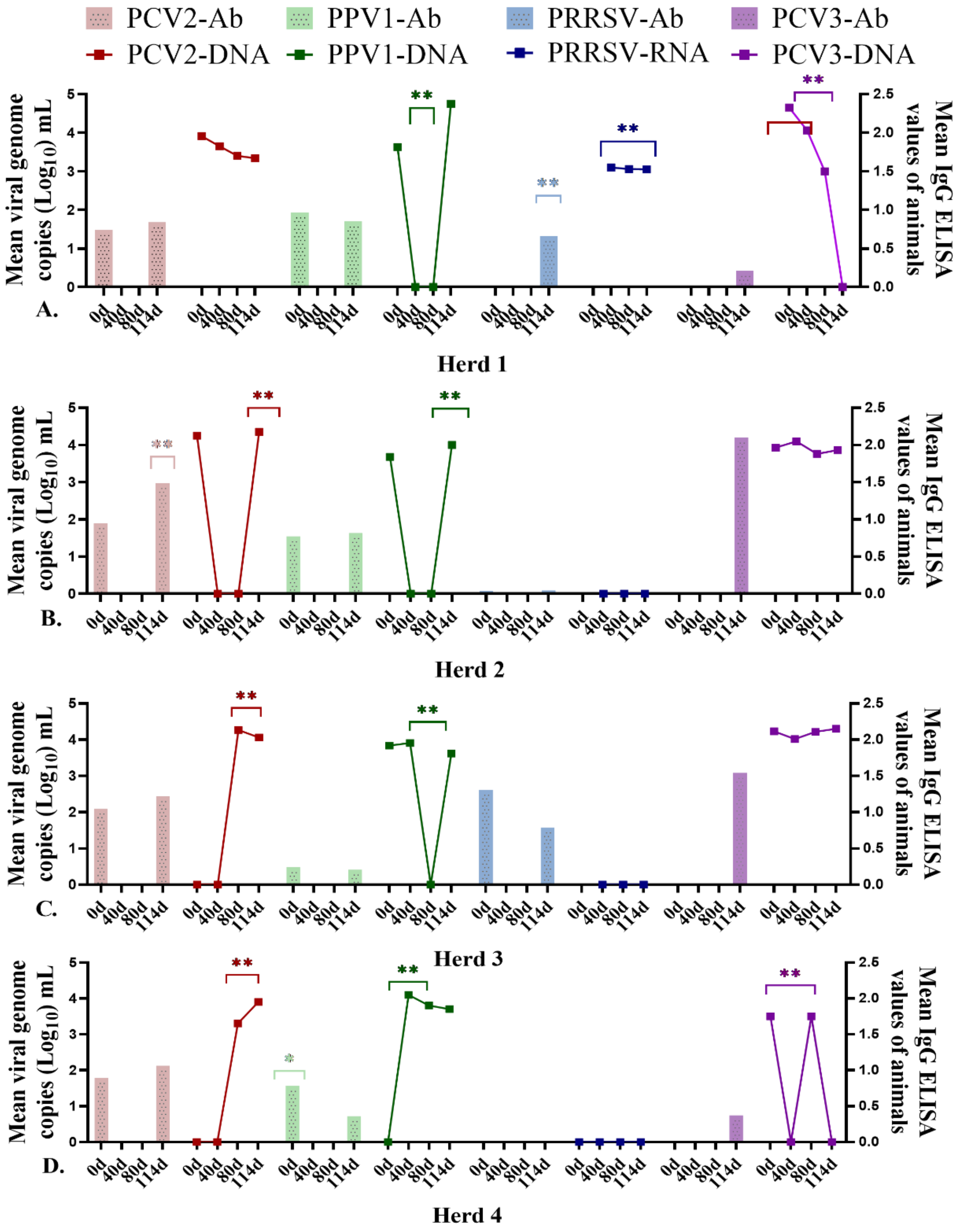

Figure S3. Viral circulation during pregnancy phase across individual herds, illustrating Ab levels (bars) and viremia (symbols). The viruses' color codes are PCV2 in red, PPV1 in green, PRRSV in blue, and PCV3 in purple. The figure is divided into four panels, with each panel representing a different herd: A. Herd 1 (H1), B. Herd 2 (H2), C. Herd 3 (H3), D. Herd 4 (H4). Asterisks indicate level of significance: * $p < 0.05$ suggests significant differences, and ** $p < 0.01$ indicates higher significant differences between the samplings during PP. **Key Findings:** PCV2: Significant differences in Ab titers were observed between insemination and delivery ($p < 0.001$) in H2. Additionally, H1 showed significant differences in viral load at farrowing ($p < 0.05$). PCV3: H2 and H3 showed the highest Ab levels ($p < 0.01$). The mean viral load for PCV3 showed significant differences between sampling points in H1 and H4 ($p < 0.01$). PPV1: Differences in Ab levels were noted in H1 and H2 at both insemination and delivery ($p < 0.01$). H4 demonstrated a significant decrease in Ab levels from insemination to delivery ($p < 0.01$), while H3 exhibited differences in Ab titers compared to all other herds at both insemination and delivery. Viremia for PPV1 showed significant differences between sampling points across all farms ($p < 0.01$). PRRSV: Differences between sampling points in H1 ($p < 0.01$).

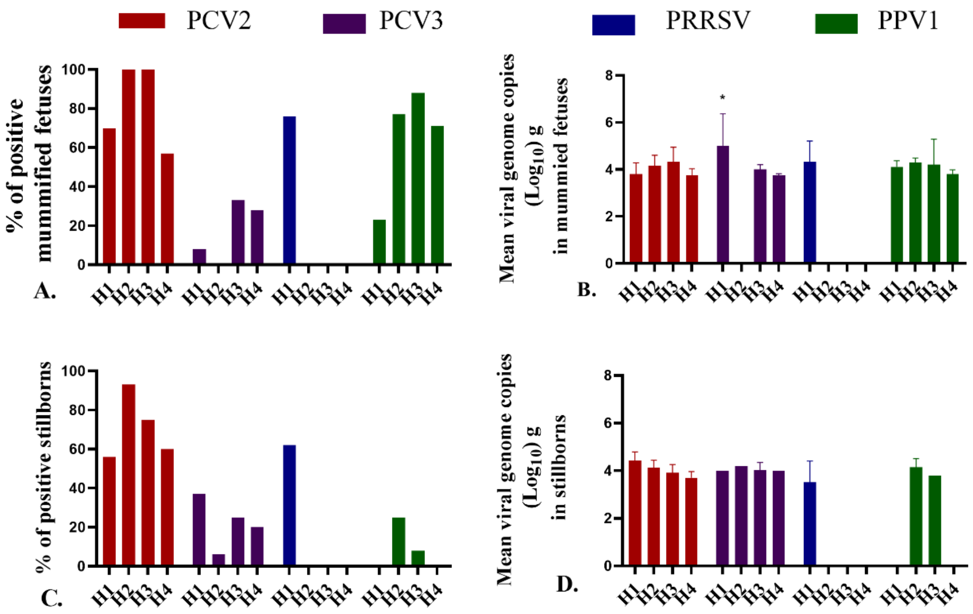

Figure S4. Viral detection rates and viral loads (Log10 copies/g) in mummified fetuses and stillborn piglets from individual herds. This analysis focuses on four primary reproductive viruses, each represented by a distinct color: PCV2 in red, PPV1 in green, PRRSV in blue, and PCV3 in purple. The data is organized into four separated panels: A. Percentage of viral detection in mummified fetuses; B. Viral load (log10 copies/ml) in mummified fetuses; C. Percentage of viral detection in stillborn piglets; D. Viral load (log10 copies/ml) in stillborn piglets. Asterisks * indicated significant differences, where $p < 0.05$ denotes significant differences.

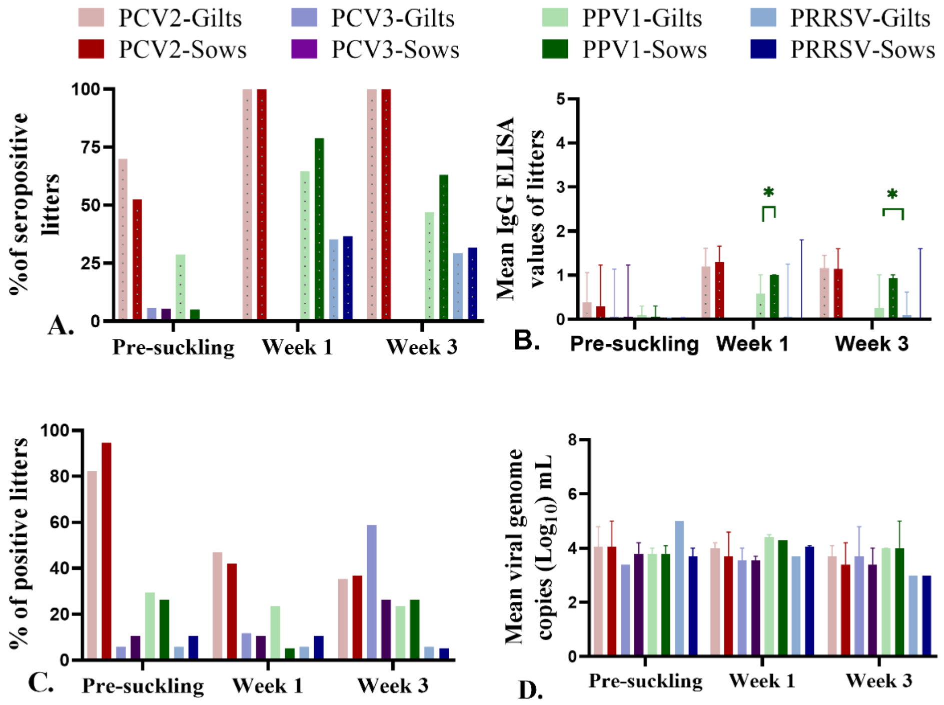

Figure S5. Boxplot depicting viral loads in fetuses for PCV2, PCV3, PPV1, and PRRSV based on the type of infection: mono-infection (green), dual infection (red), and triple infection (blue). The panel keys are A. PCV2, B. PCV3, C. PPV1, and D. PRRSV. Asterisks * indicated significant differences, where $p < 0.05$ denotes significant differences.

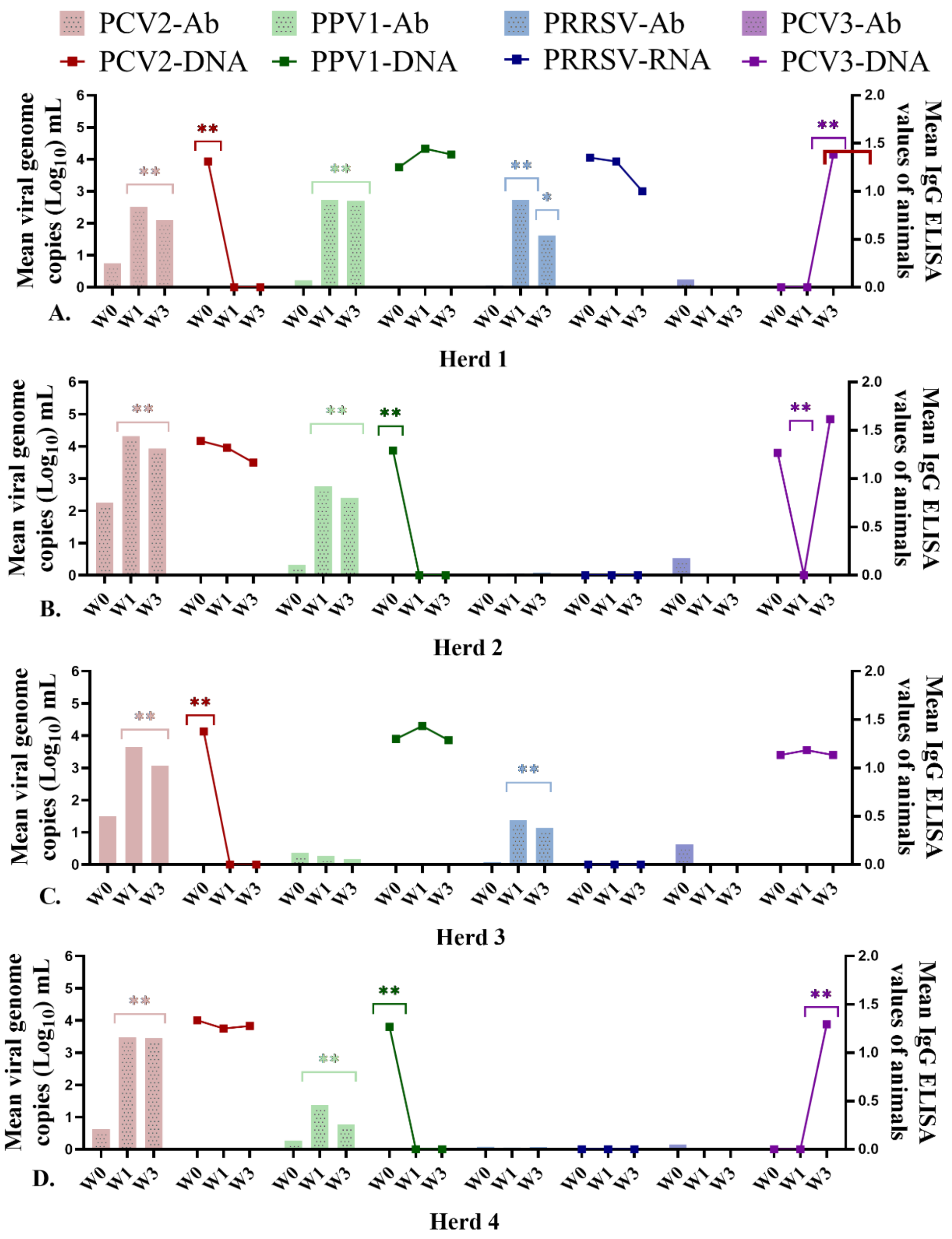

Figure S6. Viral circulation during the lactation phase (LP) in litters from gilts and sows. This analysis covers four primary reproductive viruses, each represented by a distinct color: PCV2 (red), PPV1 (green), PRRSV (blue), and PCV3 (purple). The figure is divided into four panels, each illustrating a different aspect of viral circulation: A. Percentage of seropositivity litters from gilts and sows; B. Mean IgG ELISA values of litters; C. Percentage of viral positive litters from sows or gilts; D. Mean viral genome copies (log10) mL). * p <0.05 significant differences between litters from gilts and sows, ** indicates highly significant differences at p < 0.01. **Key findings:** The IgG antibody levels for PPV1 were significantly higher in litters from multiparous sows during weeks 1 and 3 of the nursery phase.

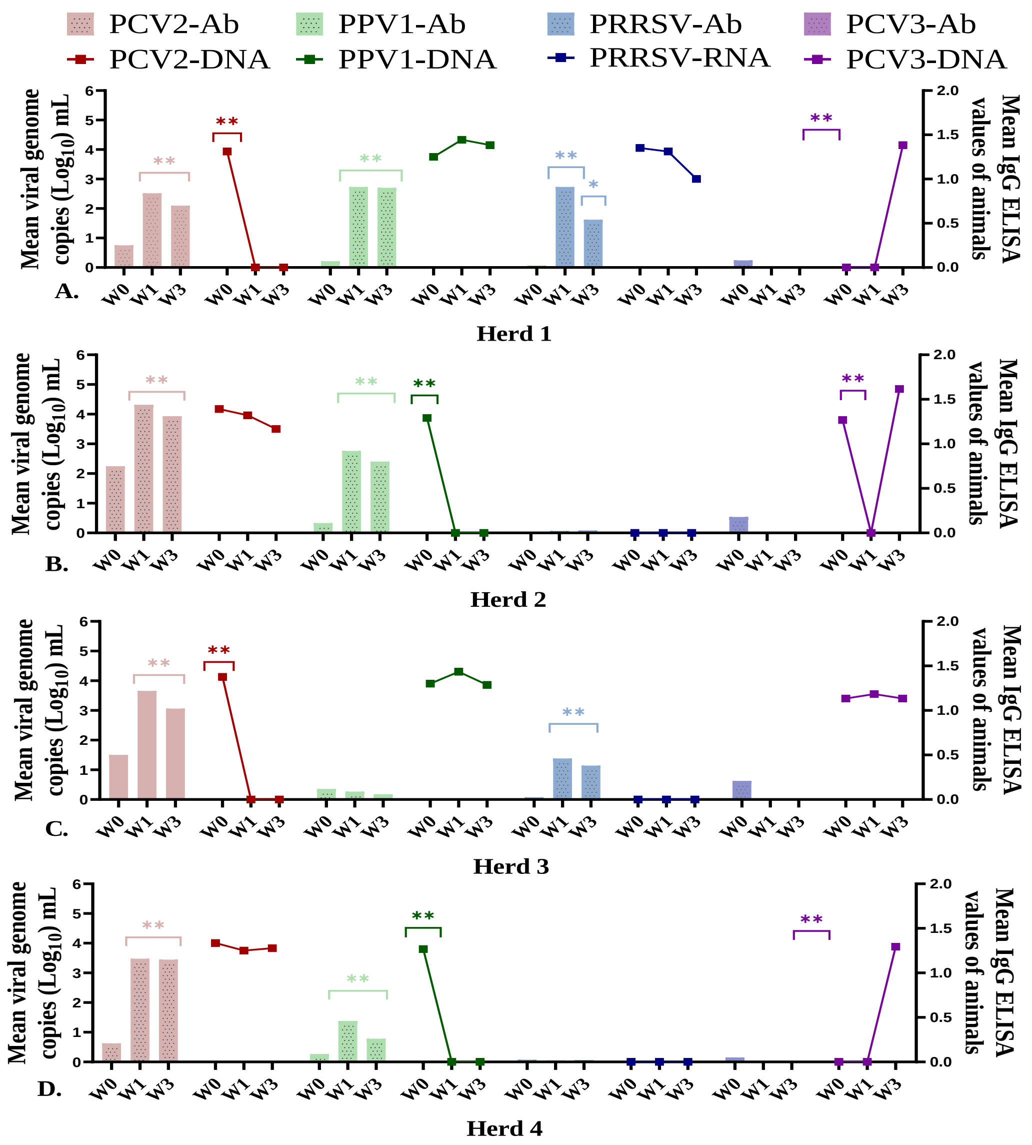

Figure S7. Viral circulation during the lactation phase (LP) across individual herds, showing Ab levels (bars) and viremia (symbols). The colors represent viruses: PCV2 in red, PPV1 in green, PRRSV in blue, and PCV3 in purple. The figure is divided into four panels, each representing a different herd: A. Herd 1 (H1), B. Herd 2 (H2), C. Herd 3 (H3), D. Herd 4 (H4). An asterisk * denotes significant differences at $p < 0.05$, while ** indicates highly significant differences at $p < 0.01$ between the sampling points during LP. **Key findings: PCV2**: Significant differences were observed in antibody (Ab) levels between farms, with H1 consistently showing the lowest levels across all sampling points ($p < 0.01$). There were significant differences in Ab levels between pre-suckling and weeks 1 and 3 (p < 0.01) but not between weeks 1 and 3. Significant differences in viral load between the herds were observed during weeks 1 and 3 ($p < 0.01$). Within-farm, differences between sampling points were identified in H1 and H3 ($p < 0.001$). **PCV3**: Significant differences in viral loads were observed in week 1 and week 3 in H1, H2, and H4 ($p < 0.01$). **PPV1**: Ab levels were notably high during week 1 of life ($p < 0.01$), and these levels were maintained in week 3 in H1 and H2. In H4, Ab levels increased during week 1 but declined below the detection limit by week 3 ($p < 0.01$). In H3, the litter did not seroconvert in any evaluated weeks. **PRRSV**: Higher Ab titers were measured during week 1 of life, which declined by week 3 in H1 and H3 ($p < 0.01$). Significant differences ($p = 0.04$) were found between the evaluated weeks, with lower levels at weaning.

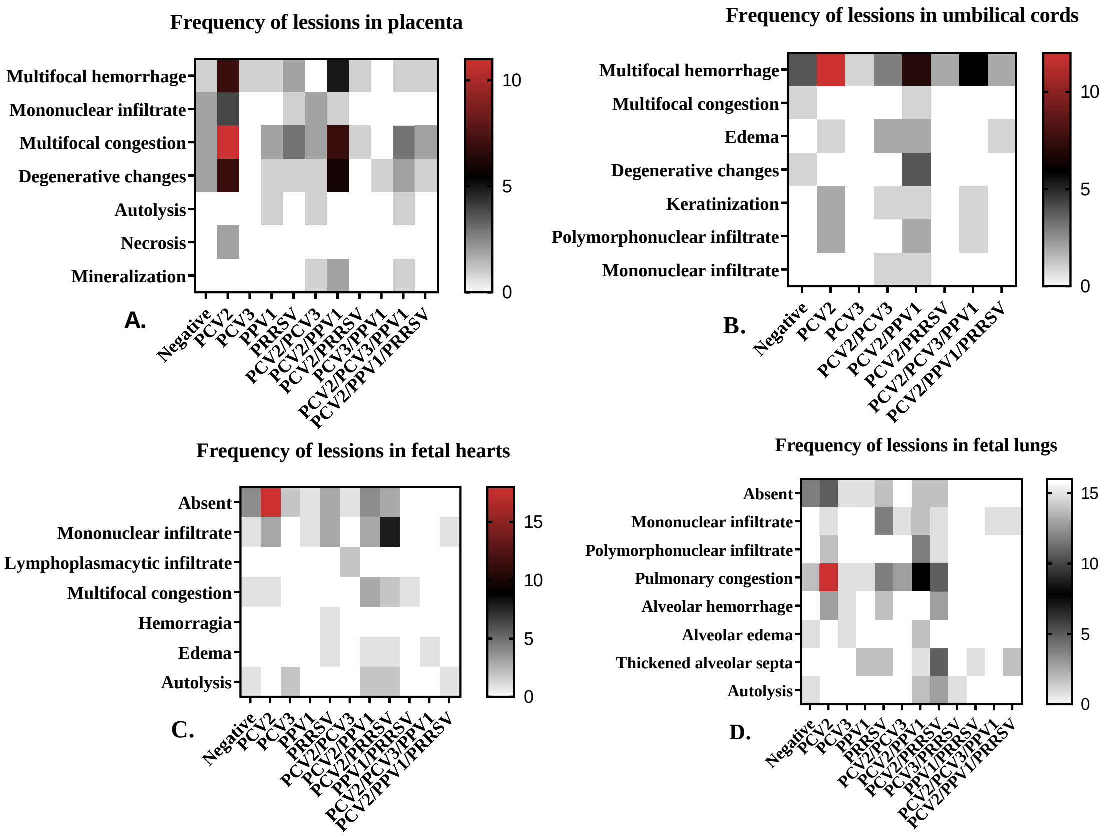

Figure S8. Heat map that illustrates the frequency of histopathological lesions found in tissues collected at delivery from gilts, sows, and fetuses, along with their association with different types of infection. Specifically, it showcases A. Placental lesions, B. Umbilical cord lesions, C. Fetal heart lesions, and C. Fetal lung lesions. The lateral ranges from 0 to 15, indicating the frequency (number) of lesions associated with each type of infection. Red represents the highest frequency of lesions, black indicates an average frequency, and white signifies the absence of lesions.

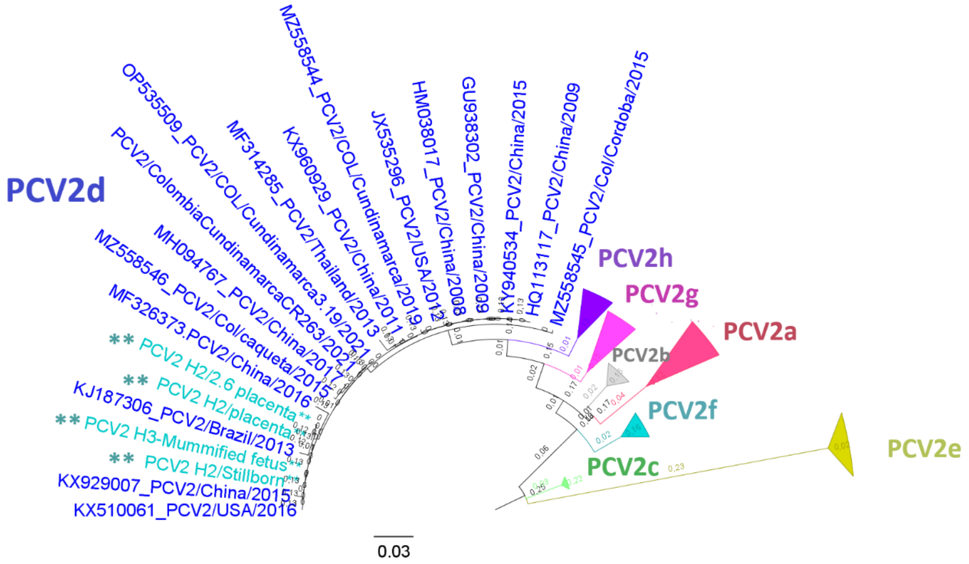

(**A**)

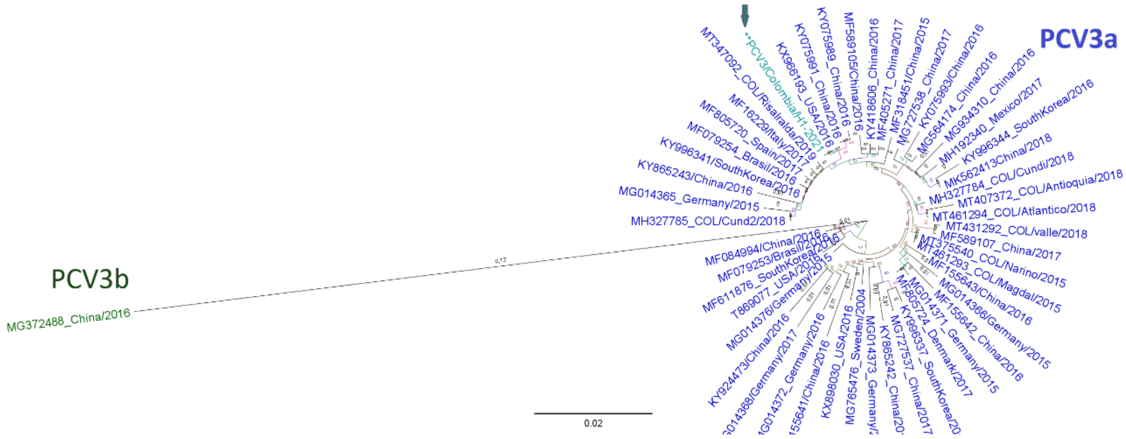

(B)

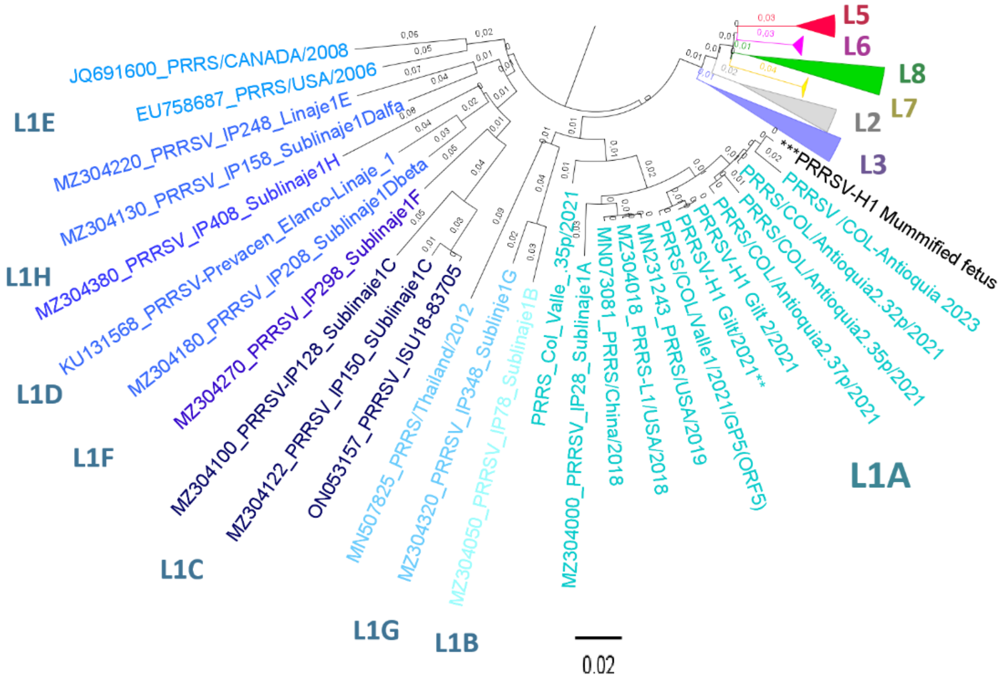

(C)

Figure S9. Maximum likelihood (ML) phylogenetic trees for PCV2-ORF2, PCV3-ORF2, and PRRSV-ORF5 were estimated based on the alignment of the nucleotide sequences. The ML phylogenetic trees were constructed using the Tamura-Nei model with a gamma distribution. The tree topology was evaluated with 1,000 bootstrap replicates. An asterisk * indicates sequences generated in this study. Panel A shows the PCV2 phylogenetic tree, Panel B shows the PCV3 phylogenetic tree, and Panel C shows the PRRSV phylogenetic tree.

Table S1. Characteristics of the evaluated pig herds in this study, including the reproductive parameters of the monitored gilts (primiparous sows) and sows (multiparous sows) over time, as well as details of the collected samples.

	**Herd 1 (H1)**	**Herd 2 (H2)**	**Herd 3 (H3)**	**Herd 4 (H4)**
**Province**	**Antioquia**	**Cundinamarca**	**Atlántico**	**Cundinamarca**
Sow inventory	1200	550	1666	1700
Gilts PCV2 vaccination protocol	175 days (CircoFLEX®)	130-140 days (CircoFLEX®)	170 days (PORCILIS®)	175 days (CircoFLEX®)
Gilts PPV1 vaccination protocol	170-190 days (ERYSENG®)	155-165 days (Farrowsure®)	145-165 days (Farrowsure®)	170-190 days (ERYSENG®)
Reproductive parameters of herds				
Total born piglets index	17.0	15.8	15.0	17.3
Piglets born alive index	12.0	13.3	12.6	16.0

% Mummies	20.0	5.6	6.6	4.3
% Stillborn	9.4	10.1	8.8	3.5
% Preweaning mortality (PWM)	4.1	7.6	NR	0.7
Number and type of samples collected				
Primiparous and multiparous serum	40	40	39	38
Placenta	10	10	9	8
Umbilical cord	10	10	9	8
Colostrum	10	10	9	8
Litters (serum)	10	10	8	8
Mummies	34	9	9	6
Stillborn	16	16	12	5

NR: not reported.

Table S2. List of primers used in the present study for virus detection.

Primer	Name	Sequence 5´to 3´	Reference
B actin	pBactin-F<br>pBactin-P	CTTCTGCCATTTTCCTAGGACTTTT<br>AACACCTAGTCAGAAAGGCAAACA	[18]
PRRSV	PRRSVF<br>PRRSVR<br>Probe PRRVS	ATRATGRGCTGGCATTCC<br>ACACGGTCGCCCTAAT-TG<br>FAM- TGTGGTGAATGGCACTGATTGACA -TAMRA	[19]
PCV2	PCV2abF PCV2abR<br>ProbePCV2a<br>ProbePCV2b/d	GCAGGGCCAGAATTCAACC<br>GGCGGTGGACATGATGAGA<br>FAM-GGGGACCAACAAAATCTCTATACCCTTT-BHQ<br>Cy5-CTCAAACCCCCGCTCTGTGCCC-BHQ	[20]
PCV3	PCV3F<br>PCV3R<br>Probe PCV3	AGACGACCCTTATGCGGAAA<br>AACGGTGGGGTCATATGTGTTG<br>FAM-CTCACCCAGGACAAAGCCTCTTCTT-BHQ	[22]
PPV1	PPV1F<br>PPV1R<br>Probe PPV1	GAAGACTGGATGATGACAGATCCA<br>TGCTGTTTTTGTTCTTGCTAGAGTAA<br>FAM-AATGATGGCTCAAACCGGAGGAGA-BHQ	[21]
PPV2	PPV2-F<br>PPV2-R	AGCTCTGCGACAAGTGGG<br>GTCTACGGCCTGCAAGAA	[24]
PPV3	PPV3-F<br>PPV3-R	CAYGAYGAACGGTACGATGAAAT<br>GCGGTAAAACCTGTGAWAWTTGAAC	[25]
PPV4	PPV4-F<br>PPV4-R	TATGTGGGCTGGGCAAGGAATGTC<br>GTTGCGGAATGCTATCAGGCTCTT	[26]
PPV5	PPV5-F<br>PPV5-R	GCATTGGTGTGTGTCTGTGTCC<br>GTGGCACATTTGTACATGGGAG	[26]
PPV6	PPV6-F<br>PPV6-R	GGCTTCATAATCCCTCCAAAACCT<br>GCTCATCTTCCTCTTGTTTCTCCTG	[23]
PPV7	PPV7-F<br>PPV7-R	GAGGCGGTGATGGAGCAGAT<br>CTCCAGGACCACCACATCCC	[27]
PPV8	PPV8-outF PPV8-outR PPV8-inF PPV8-inR	TGTTGGTTTGCACCTAGCG<br>TGATGAGATGGTGGAACGC<br>TCCAAGTTGCCCTAGACAGC<br>GCCTCGTACATGTGGACCTC′	[28]
PCV4	PCV4-F<br>PCV4R	GTTTTTCCCTTCCCCCACATAG<br>ACAGATGCCAATCAGATCTAGGTAC	[29]

Table S3. Standarized Real-time PCRs details for identifiying and quantifiyng PCV2, PCV3, PPV1, and PRRSV in this study.

Virus	LoD: Linear dynamic range	LoD: Log $_{10}$ copies ranging	Ct values ranging	Efficiency	R^2
PCV2	6 x10^9- 6 x10^1	9.8-1.8	12.5 - 37	91.3%	0.98
PCV3	7 x 10^8- 7 x 10^1	9.77-2.77	12.7 - 36	106%	0.99
PPV1	2 x10^8-2 x10^1	8.4-1.4	11.5 - 34	104%	0.99
PRRSV	3 x10^9-3 x10^1	10-2	14 - 36.5	105%	0.99

LoD: limit of detection, R^2: linear correlation index.

Table S4. ELISA results and qPCR viral loads for PCV2, PCV3, PPV1, and PRRSV at insemination and delivery across the four evaluated herds (H1 to H4).

Herd	Serology (Mean IgG ELISA Values and number of seropositive)							Viral load (mean viral genome copies Log 10 mL and number of positive)							
	PCV2		PCV3	PPV1		PRRSV		PCV2		PCV3		PPV1		PRRSV	
	Insemination	Delivery	Delivery	Insemination	Delivery	Insemination	Delivery	Insemination	Delivery	Insemination	Delivery	Insemination	Delivery	Insemination	Delivery
**H1**	0.77±0.17 (10/10)	0.84±0.3 (10/10)	0.21±0.4 (3/10)	0.97±0.3 [a] (9/10)	0.86±0.3 [a] (9/10)	0/10	0.66±0.23 **	3.91±0.3 (6/10)	3.34±0.35 (7/10)	4.65±1.6 (3/10)	0/10	3.63±0.16 (4/10)	4.75±0.35 * (2/10)	0/10	3.05±0.1 (4/10)
**H2**	0.95±0.09 (10/10)	1.49±0.1 **/a (10/10)	2.1±1 [a] (10/10)	0.77±0.4 [a] (7/10)	0.81±0.3 [a] (8/10)	0/10	0/10	4.25±0.78 (8/10)	4.35±1.03 (9/10)	3.93±0.11 (3/10)	3.8±0.15 (4/10)	3.69±0.17 (5/10)	4±0.28 (2/10)	0/10	0/10
**H3**	1.05±0.2 (10/10)	1.22±0.36[a] (10/10)	1.43±1.3[a] (8/10)	0.24±0.1 (1/10)	0.2 (1/10)	1.31±0.59 (10/10)	0.79±0.78 (5,9)	**0/10**	4.06±0.8** (8/10)	4.23±0.73 (6/10)	4.3±0.0 (1/10)	3.84±0.12 (10/10)	3.62±0.16 *(5/10)	0/10	0/10
**H4**	0.89±0.20 (10/10)	1.03±0.29 (8/10)	0.42±0.7 (3/8)	0.79±0.3[a] (9/10)	0.36±0.1 * (4/10)	0/10	0/8	**0/10**	3.9±0.41** (8/8)	3.5±0.05 (3/10)	0/8	0/10	3.7±0.2 *(4/8)	0/10	0/8
**Total**	**0.91±0.33 (40/40)**	**1.16±0.36 (38/38)**	**1.06±1.1 (24/38)**	**0.7±0.39 (26/40)**	**0.57±0.36 22/38**	**0.67±0.75 (10/40)**	**0.72±0.52 (14/38)**	**4.10±0.61 (14/40)**	**3.95± 0.7(33/3)**	**4.10±0.9 (15/40)**	**3.87± 0.25(5/38)**	**3.75±0.16 (19/40)**	**3.88±0.4 (13/38)**	**0/40**	**3.05±0.1 (4/38)**

± denotes standard deviation, * $p < 0.05$: Significant differences within farms between insemination and delivery ** $p < 0.01$: Highly significant differences, [a] Denotes differences between farms. H1: Herd 1, H2: herd 2, H3: herd 3, H4: herd 4.

Table S5. Mean Viral loads (log 10 copies/mL) for PCV2, PCV3, PPV1, and PRRSV in different types of infection (mono and coinfections) detected during the pregnancy phase (PP). .

Pregnancy Phase	Infection type	PCV2	PCV3	PPV1	PRRSV
**Insemination**	PCV2 (n=4)	4.45 (±0,9)*			
	PCV3 (n=3)		4.1 (±0,2)		
	PPV1 (n=6)			3.7 (±0,18)	
	PCV2/PCV3 (n=3)	4.3 (±0,4)*	4 (±1.5)		
	PCV2/PPV1 (n=4)	3.6 (±0,38)		3.6 (±0,15)	
	PCV3/PPV1 (n=6)		4 (±0,7)	3.85 (±0,12)	
	PCV2/PCV3/PPV1 (n=3)	3.8 (±0,06)	3.8 (±0,14)	3.84 (±0,02)	
**40 days**	PCV3 (n=5)		4.1 (±0,2)		
	PPV1 (n=2)			4.3 (±0,2)*	
	PRRSV (n=2)				3 (±0,0)
	PCV2/PRRSV (n=2)	4.2 (±0,0)			3.2 (±0,28)
	PCV3/PPV1 (n=5)		4 (±0,2)	3.8 (±0,12)	
	PCV3/PRRSV (n=3)		4 (±0,4)		3 (±0,0)
	PCV2/PCV3/PRRSV (n=1)	3 (±0,0)	4.3 (±0,0)		3.4 (±0,0)
**80 days**	PCV2 (n=7)	4.2 (±0,06)			
	PCV3 (n=10)		3.5 (±0,3)		
	PPV1 (n=1)			3.7 (±0,0)	
	PRRSV (n=6)				3 (±0,16)
	PCV2/PCV3 (n=7)	3.9 (±0,56)	4 (±0,5)		
	PCV2/PPV1 (n=3)	3.4 (±0,4)		3.8 (±0,15)	
	PCV3/PRRSV (n=1)		3.5 (±0,0)		3 (±0,0)
**Delivery**	PCV2 (n=15)	3.6 (±0,6)			
	PPV1 (n=2)			4.35 (±0,9)*	
	PCV2/PCV3 (n=3)	5.4 (±0,5)*	4 (±0,28)		
	PCV2/PPV1 (n=8)	4.2 (±0,4)*		3.7 (±0,13)	
	PCV2/PRRSV (n=3)	3 (±0,46)			3 (±0,11)
	PCV2/PCV3/PPV1 (n=2)	4.7 (±1)*	3.8 (±0,19)	4 (±0,28)*	
	PCV2/PPV1/PRRSV (n=1)	3.8 (±0,0)		4.5 (±0,0)	3 (±0,0)

± denotes standard deviation, *p < 0.05: Significant differences between viral load according to mono-infection and coinfection.

Table S6. Viral load and positivity for PCV2, PCV3, PPV1, and PRRSV in the placenta, umbilical cords, and colostrum across the four herds evaluated (H1 to H4) during the farrowing phase (FP).

Farrowing samples	PRRSV				PCV2				PCV3				PPV1			
	H1	H2	H3	H4	H1	H2	H3	H4	H1	H2	H3	H4	H1	H2	H3	H4
**Placenta**	3.3 ±0.6 (5/10)	-	-	-	3.8 ±0.3 (6/10)	4.5±0.5 (10/10)	4.3±0.4 (9/9)	4±0.2 (2/8)	0	3.7±0.02 (3/10)	3.9±0.2 (3/9)	3.9±0.3 (2/8)	3.5±0.03 (3/10)	4.2±0.4 (5/10)	3.9±0.1 (4/9)	3.8±0.1 (4/8)
**Umbilical cord**	3.2 ±0.4 (4/10)	-	-	-	4 ±0.24 (9/10)	4.8±0.6 (10/10)	4.1±0.3 (8/9)	4±0.16 (5/8)	0	3.7±0.02 (4/10)	3.8±0.1 (3/9)	4.1±0.1 (2/8)	4.1±0.8 (3/10)	4.4±0.6(5/10)	3.9±0.1 (3/9)	3.9±0.1 (4/9)
**Colostrum**	3.2 ±0.4 (3/10)	-	-	-	3.8 ±0.3 (5/10)	4.2±0.3 (10/10)	4.3±0.7 (8/9)	3.8±0. 1(4/8)	0	4±0.4 (6/10)	3.9±0.2 (3/9)	4.3±0.9 (2/8)	4.5±1 (2/10)	4.1±0.6 (4/10)	3.7±0.2(4/9)	3.9±0.1 (3/9)

± denotes standard deviation, - : denotes herds negative, H1: Herd 1, H2: Herd 2, H3: Herd 3, H4: Herd 4.

Table S7. Number and percentage of infection types (mono and coinfections) detected in fetal samples (mummies and stillborns).

Fetuses	Negative n (%)	PCV2 n (%)	PCV3 n (%)	PPV1 n (%)	PRRSV n (%)	PCV2/ PCV3 n (%)	PCV2/ PPV1 n (%)	PCV2/ PRRSV n (%)	PCV3/ PRRSV n (%)	PPV1/ PRRSV n (%)	PCV2/ PCV3/PPV1 n (%)	PCV2/ PPV1/PRRSV n (%)	Total
**Mummies**	1 (1.7)	6(10.3)	2 (3.4)	2 (3.4)	6 (10.3)	2 (3.4)	17 (29.3)	13 (22.4)	1 (1.7)	1 (1.7)	2 (3.4)	5 (8,6)	58
**Stillborns**	6 (12.2)	22 (44,9)	2 (4)	0 (0)	5 (15,2)	4 (8.1)	5 (15,2)	5 (15,2)	0 (0)	0 (0)	0 (0)	0 (0)	49
**Total**	7 (6.5)	28 (26.1)	4 (3,7)	2 (1,8)	11 (10,2)	6 (5,6)	22 (20,5)	18 (16,8)	1 (0.9)	1 (0.9)	2 (1,8)	5 (4,6)	107

Table S8. Frequency of PRRSV, PCV2, PCV3, and PPV1 in mummified fetuses and stillborns across different herds.

Herd	Sample	N	PRRSV n (%)	PCV2 n (%)	PCV3 n (%)	PPV1 n (%)
1	Mummies	34	26/34 (76.47%)	24/34 (70.58%)	3/34(8.82%)	8/34(23.52%)
	Stillborns	16	10/16 (62.50%)	9/16 (56.25%)	1/16 (6.25%)	0/16 (0%)
2	Mummies	9	0/9 (0%)	9/9 (100%)	0/9 (0%)	7/9(77.77%)
	Stillborns	16	0/16 (0%)	15/16 (93.75%)	1/16 (6.25%)	4/16 (25%)
3	Mummies	9	0/9 (0%)	8/9 (88.88%)	3/9 (33.33%)	8/9(88.88%)
	Stillborns	12	0/12 (0%)	9/12 (75%)	3/12 (25%)	1/12 (8.33%)
4	Mummies	6	0/6 (0%)	4/6 (66.66%)	2/6 (33.33%)	5/6(83.32%)
	Stillborns	5	0/5 (0%)	3/5 (60%)	1/5 (20%)	0/5 (0%)
Total		107	36/107 (33.64%)	81/107 (75.70%)	14/107 (13.08%)	33/107 (30.84%)

Table S9. Mean Viral loads (log 10 copies/mL) established for different types of infections in fetal samples.

Infection type	PCV2	PCV3	PPV1	PRRSV
PCV2 (n=28)	4 (+/- 0,44)	0	0	0
PCV3 (n=4)		4 (+/- 0,44)		
PPV1 (n=2)			3,85 (±0,07)	
PRRSV (n=11)				3,9 (±1,09)
PCV2/PCV3 (n=6)	3,9 (±0,33)	3,95 (±0,26)		
PCV2/PPV1 (n=22)	4,5 (±0,33)*		4 (±0,32)	
PCV2/PRRSV (n=18)	4,1 (±0,5)			4,2 (±0,67)
PCV3/PRRSV (n=1)	7,5 (±0)			4,7 (±0)
PPV1/PRRSV (n=1)	4,5 (±0)			4,7 (±0)
PCV2/PCV3/PPV1 (n=2)	4,05 (±0,32)	3,8 (±0,23)	5 (±1,35)	
PCV2/PPV1/PRRSV (n=5)	3,5 (±0,02)		4,3 (±0,31)	3 (±1,3)

± denotes standard deviation, *p < 0.05: Significant differences between viral load according to mono-infection and coinfection.

Table S10. Multivariate analysis of associations between detected viral infections (PCV2, PCV3, PPV1, and PRRSV) in fetuses (dependent variables) and coinfection status, parity, and vaccination against PCV2 and PPV1 (independent variables).

Independent variables	Dependent variables			
	**PCV2 positive fetus**	**PCV3 positive fetus**	**PPV1 positive fetus**	**PRRSV positive fetus**
**Parity status (PS or MS)**	NS	NS	NS	NS
**PCV2 vaccination protocol**	NS	NS	NS	NS
**PPV1 vaccination protocol**	NS	NS	NS	NS
**PCV2-positive fetus**	-	NS	$p < 0.01$	NS
**PCV2-negative fetus**	NS	NS	NS	$p = 0.06$
**PCV3-positive fetus**	NS	-	NS	NS
**PCV3-negative fetus**	NS	NS	NS	$p = 0.02$
**PPV1-positive fetus**	NS	NS	-	NS
**PPV1-negative fetus**	NS	NS	NS	$p = 0.04$
**PRRSV-positive fetus**	NS	NS	NS	-
**PRRSV-negative fetus**	$p = 0.04$	$p = 0.05$	$p = 0.06$	NS

PS: primiparous sows. MS: multiparous sows. NS: Non-significant differences.

Table S11. Results from ELISA and viral loads for PCV2, PCV3, PPV1, and PRRSV during the lactating phase (LP) in each evaluated herd.

Herd	Serology (Mean IgG ELISA Values and number of seropositive)									
	PCV2			PCV3	PPV1			PRRSV		
	PS	W1	W2	PS	PS	W1	W3	PS	W1	W3
H1	0.25±0.1 (3/10)	0.83±0.2* (10/10)	0.7±0.3* (10/10)	0/10	0/10	0.91±0.30^{a} (9/10)	0.89±0.2^{a} (9/10)	0/10	0.91±0.25 (10/10)	0.54±0.1* (10/10)
H2	0.75±0.3 (10/10)^{a}	1.44±0.1* (10/10)	1.3±0.1* (10/10)	0.18±0 (1/10)	0.11±0.1 (2/10)	0.92±0.17^{a} (10/10)	0.80±0.3^{a} (10/10)	0/10	0/10	0/10
H3	0.50±0.22 (7/8)^{a}	1.22±0.4* (8/8)	1.02±0.3* (8/8)	0.21±0 (1/8)	0.12±0.1 (2/8)	0.1±0.08 (1/8)	0.06±0 (0/8)	0/8	0.88±0.77 (4/8)	0.56±0.6* (4/8)
H4	0.21±0.1 (1/8)	1.15±0.3* (8/8)	1.1±0.2* (8/8)	0/8	0.1±0.1 (1/8)	0.46±0.19 (6/8)	0.26±0.1** (2/8)	0/8	0/8	0/8
Total	0.44±0.31 (21/36)	1.16±0.3 (36/36)	1.04±0.3 (36/36)	0.20±0.4 (2/36)	0.1±0.08(5/36)	0.63±0.39 (26/36)	0.54±0.4 (21/36)	0/36	0.90±0.41 (14/36)	0.55±0.3 (14/36)

Herd	Viral load (mean viral genome copies Log_{10} mL and number of positive)											
	PCV2			PCV3			PPV1			PRRSV		
	PCS	W1	w3	PS	W1	W3	PS	W1	W3	PC	Sem1	Sem3
H1	3.93±0.4* (6/10)	0/10	0/10	0/10	0/10	4.15±0.6 (2/10)	3.75±0.35 (2/10)	4.33±0.3* (3/10)	4.15±0.4^{a} (6/10)	4.05±0.6 (4/10)	3.93±0.2 (3/10)	3.08±0.2 (5/10)
H2	4.1±0.25 (10/10)	3.96±0.46 (9/10)	3.5±0.26** (7/10)	3.8±0.5 (2/10)	3.8* (1/10)	4.85±1.6*^{a} (2/10)	3.88±0.15 (4/10)	(0/10)	0/10	0/10	0/10	0/10
H3	4.1±0.5* (8/8)	0/8	0/8	3.4±0.0 (1/8)	3.55±0.38* (4/8)	3.4±0.24* (4/8)	3.9±0.14 (2/8)	4.3±0.0 (1/8)	3.86±0.1^{a} (3/8)	0/8	0/8	0/8
H4	4.01±0.5 (8/8)	3.75±0.02 (7/8)	3.8±0.3 (6/8)	0/8	0/8	3.88±0.5* (7/8)	3.8±0.01 (2/8)	0/8	0/8	0/8	0/8	0/8
Total	4.07±0.42 (10/36)	4 ±0.4 (16/36)	3.65±0.3 (13/36)	3.66±0.4 (3/36)	3.7±0.3 (5/8)	3.92±0.71 (8/36)	3.84±0.15 (10/36)	4.33±0.18 (4/36)	4.05±0.34 (9/36)	4.05±0.6 (4/36)	3.93±0.2 (3/10)	3.08±0.2 (5/10)

± denotes standard deviation, *$p < 0.05$: Significant differences within farms between insemination and delivery, ** $p < 0.01$: Higher significant differences, ^{a} Denotes differences between farms. H1: Herd 1, H2: herd 2, H3: herd 3, H4: herd 4. PS: Pre-suckling, W1: Week 1, W3: Week 3.

Table S12. Average viral loads (log 10 copies/mL) for different types of infections during the lactation phase (LP).

Piglet Aged	Infection type	PCV2	PCV3	PPV1	PRRSV
Pre-suckling	PCV2 (n=20)	4.1 (±0,5)			
	PPV1 (n=1)			3.4 (±0,0)	
	PCV2/PCV3 (n=1)	3.4 (±0,0)	3.4 (±0)		
	PCV2/PPV1 (n=6)	4.15 (±0,39)		3.8 (±0,08)	
	PCV2/PRRSV (n=3)	4 (±0,1)			3.7 (±0,25)
	PPV1/PRRSV (n=1)			3.5 (±0,0)	5 (±0,0)
	PCV2/PCV3/PPV1 (n=2)	4.2 (±0,0)	3.8 (±0,56)	3.95 (±0,21)	
Week 1	PCV2 (n=16)	4 (±0,41)			
	PCV3 (n=4)		3.55 (±0,38)		
	PPV1 (n=4)			4.4 (±0,11)	
	PRRSV (n=2)				4 (±0,21)
	PPV1/PRRSV (n=1)			4 (±0,0)	3.7 (±0,0)
Week 3	PCV2 (n=7)	3.4 (±0,001)			
	PCV3 (n=7)		3.7 (±0,53)		
	PPV1 (n=5)			4 (±0,47)	
	PCV2/PCV3 (n=6)	4 (±0,28)	3.85 (±0,9)		
	PCV3/PPV1 (n=2)		4 (±0,8)	3.8 (±0,0)	
	PPV1/PRRSV (n=2)			4 (±0,0)	3 (±0,0)

± denotes standard deviation, *$p < 0.05$: Significant differences between viral load according to mono-infection and coinfection.

Table S13. Frequency of histopathological findings in maternal and fetal tissues established according to different types of infection (mono and coinfections).

sample	Lession	Negative	PCV2	PCV3	PPV1	PRRSV	PCV2/ PCV3	PCV2/ PPV1	PCV2/ PRRVS	PPV1/ PRRSV	PCV3/ PPV1	PCV2/ PCV3/PPV1	PCV2/ PPV1/PRRSV
placenta	Multifocal hemorrhage	1	7	1	1	2	-	5	1	-	-	1	1
	Mononuclear infiltrate	2	4	-	-	1	2	1	-	-	-	-	-
	Multifocal congestion	2	11	-	2	3	2	7	1	-	-	3	2
	Degenerative changes	2	7	-	1	1	1	6	-	-	1	2	1
	Autolysis	-	-	-	1	-	1	-	-	-	-	1	-
	Necrosis	-	2	-	-	-	-	-	-	-	-	-	-
	Mineralization	-	-	-	-	-	1	2	-	-	-	1	-
Umbilical cords	Multifocal hemorrhage	4	12	1	-	-	3	7	2	-	-	6	2
	Multifocal congestion	1	-	-	-	-	-	1	-	-	-	-	-
	Edema	-	1	-	-	-	2	2	-	-	-	-	1
	Degenerative changes	1	-	-	-	-	-	4	-	-	-	-	-
	Keratinization	-	2	-	-	-	1	1	-	-	-	1	-
	Polymorphonuclear neutrophil infiltrate	-	2	-	-	-	-	2	-	-	-	1	-
	Mononuclear infiltrate	-	-	-	-	-	1	1	-	-	-	-	-
Fetal heart	Absent	4	18	2	1	3	1	4	3	-	-	-	0
	Mononuclear infiltrate	1	3	-	1	3	-	3	8	-	-	-	1
	Lymphoplasmacytic infiltrate	-	-	-	-	-	2	-	-	-	-	-	0
	Multifocal congestion	1	1	-	-	-	-	3	2	1	-	0	0
	Hemorrhage	-	-	-	-	1	-	-	-	-	-	0	0
	Edema	-	-	-	-	1	-	1	1	-	-	1	0
	Autolysis	1	-	2	-	-	-	2	2	-	-	0	1
Fetal lung	Absent	4	5	1	1	2	-	2	2	-	-	-	-
	Mononuclear infiltrate	-	1	-	-	4	1	2	1	-	-	1	1
	Polymorphonuclear neutrophil infiltrate	-	2	-	-	-	-	4	1	-	-	-	-
	Pulmonary congestion	2	16	1	1	4	3	8	5	-	-	-	-
	Alveolar hemorrhage	-	3	1	-	2	-	-	3	-	-	-	-
	Alveolar edema	1	-	1	-	-	-	2	-	-	-	-	-
	Thickened alveolar septa	-	-	-	2	2	-	1	5	1	-	-	2
	Autolysis	1	-	-	-	-	-	2	3	-	-	-	-
Fetal liver	Absent	2	2	-	-	-	1	2	-	-	-	-	-
	Congestion	4	12	-	-	2	3	3	2	-	-	-	-
	Hematopoiesis	-	10	1	-	2	-	3	2	-	-	-	-
	Mixed inflammatory cell infiltrate	-	-	-	-	-	-	-	2	-	-	-	-
	Mononuclear infiltrate	-	1	-	-	-	-	-	-	-	-	-	-
	Hemorrhage	-	1	-	-	-	-	1	-	-	-	-	-
	Autolysis	1	2	4	2	8	-	14	18	-	1	2	6
Fetal spleen	Absent	3	3	-	-	0	2	1	-	-	-	-	-
	Lymphoid depletion	2	8	1	-	2	1	2	-	-	-	-	-
	Congestion	-	-	-	-	2	-	-	-	-	-	-	-
	Cortex and extramedullary hematopoiesis	-	12	-	-	2	1	4	-	-	-	-	-
Fetal brain	Absent	2	3	-	-	1	1	1	-	-	-	-	-
	Congestion	-	6	1	-	2	-	2	2	-	-	-	-
	Gliosis	1	5	-	-	2	1	1	2	-	-	-	-
	Mononuclear infiltrate	-	1	-	-	-	1	-	-	-	-	-	-
	Satellitosis	-	1	-	-	1	1	1	-	-	-	-	-
